# Bioengineering of functional human induced pluripotent stem cell-derived intestinal grafts

**DOI:** 10.1038/s41467-017-00779-y

**Published:** 2017-10-10

**Authors:** Kentaro Kitano, Dana M. Schwartz, Haiyang Zhou, Sarah E. Gilpin, Gregory R. Wojtkiewicz, Xi Ren, Cesar A. Sommer, Amalia V. Capilla, Douglas J. Mathisen, Allan M. Goldstein, Gustavo Mostoslavsky, Harald C. Ott

**Affiliations:** 1 Department of Surgery, Massachusetts General Hospital, Harvard Medical School, 55 Fruit St., Boston, MA 02114 USA; 2 Department of General Surgery, Changzheng Hospital, Second Military Medical University, No.415, Fengyang Road, Shanghai, 200003 China; 3 Center for Systems Biology, Massachusetts General Hospital, Richard B. Simches Research Center, 185 Cambridge St, Boston, MA 02114 USA; 4 Center for Regenerative Medicine, Boston University School of Medicine, 72 E. Concord St., Boston, MA 02118 USA; 5 Division of Thoracic Surgery, Department of Surgery, Massachusetts General Hospital, Harvard Medical School, 55 Fruit St., Founders 7, Boston, MA 02114 USA; 6 Division of Pediatric Surgery, Department of Surgery, Massachusetts General Hospital, Harvard Medical School, 55 Fruit St., Boston, MA 02114 USA; 7 Section of Gastroenterology, Department of Medicine, Boston Medical Center, 830 Harrison Ave, Boston, MA 02118 USA; 8 Harvard Stem Cell Institute, 7 Divinity Ave, Cambridge, MA 02138 USA

## Abstract

Patients with short bowel syndrome lack sufficient functional intestine to sustain themselves with enteral intake alone. Transplantable vascularized bioengineered intestine could restore nutrient absorption. Here we report the engineering of humanized intestinal grafts by repopulating decellularized rat intestinal matrix with human induced pluripotent stem cell-derived intestinal epithelium and human endothelium. After 28 days of in vitro culture, hiPSC-derived progenitor cells differentiate into a monolayer of polarized intestinal epithelium. Human endothelial cells seeded via native vasculature restore perfusability. Ex vivo isolated perfusion testing confirms transfer of glucose and medium-chain fatty acids from lumen to venous effluent. Four weeks after transplantation to RNU rats, grafts show survival and maturation of regenerated epithelium. Systemic venous sampling and positron emission tomography confirm uptake of glucose and fatty acids in vivo. Bioengineering intestine on vascularized native scaffolds could bridge the gap between cell/tissue-scale regeneration and whole organ-scale technology needed to treat intestinal failure patients.

## Abstract (web summary)

There is a need for humanised grafts to treat patients with intestinal failure. Here, the authors generate intestinal grafts by recellularizing native intestinal matrix with human induced pluripotent stem cell-derived epithelium and human endothelium, and show nutrient absorption after transplantation in rats.

## Introduction

Short bowel syndrome (SBS) is the end-stage pathology of various gastrointestinal disorders, including Crohn’s disease, mesenteric ischemia, and midgut volvulus. Affected patients develop intestinal failure when the remaining bowel no longer has sufficient absorptive capacity to allow for enteral nutritional autonomy, and patients become dependent on intravenous nutrition for maintenance.

Small bowel transplantation is now accepted worldwide as a treatment option for patients with irreversible intestinal failure^1^. In 2015, a total of 127 intestinal transplantations were performed in the U.S., but an additional 275 patients remain on the wait list due to a shortage of suitable organs^2^. Although early-term graft survival has improved over the past decade, graft failure rate at 3 years remains high at 41.9% for transplants in 2009–2010^3^. A recipient’s evoked immune response to the allograft often results in acute cellular rejection and chronic allograft enteropathy^4^. Even when effective, life-long immunosuppression has numerous complications, including opportunistic infections, renal dysfunction, and lymphoproliferative disorders^5, 6^.

Using a patient’s own primary cells or patient-derived induced pluripotent stem cells (iPSCs) to generate a subject-specific organ has enormous potential to overcome these barriers to intestinal transplantation. It has been shown that terminally differentiated cells derived from autologous iPSCs have negligible immunogenicity^7^. Additionally, iPSCs can be directed to differentiate first into intestinal progenitor cells and then into mature epithelium^8^. The combination of expandability in culture, minimal immunogenicity, and differentiation potential makes iPSCs an ideal tool for personalized regenerative therapies. Creating culture conditions in which these cells can organize into a functional whole organ is the challenge.

Current technology for engineering intestine, whether using primary intestinal stem cells^9^ or iPSCs, has focused on the cell or tissue scale. Intestinal stem cells have been shown to form 3-D organoids with crypt-villus architecture when cultured in vitro^10^, and are able to repopulate an epithelial layer when introduced via colonic enema to mice with colitis-induced mucosal injury^11^. Human iPSCs were successfully differentiated into intestinal epithelial progenitors in vitro^8^, and formed organoids with mature epithelium when injected into mouse kidney subcapsule^12^. These results suggested the potential application of cell therapy, but would be marginally applicable for treating patients with SBS, who would require *de novo* whole segments of intestine for transplantation.

Attempts to provide intestinal progenitor cells with a physical platform have been made as early as 2004, using synthetic biodegradable tubes^13^. Decellularized intestine has been used as a scaffold for epithelial regeneration by other groups, but not in a manner that resulted in whole perfusable segments^14^. However, these efforts have generated epithelialized tubular grafts to be inserted in continuity with native bowel, which would not have the absorptive capability coupled with functional vasculature needed to restore enteral nutrient absorption to patients with intestinal failure.

On the basis of our previous experience with whole-organ heart^15^, lung^16^, and kidney^17^ extracellular matrix (ECM) scaffolds, we hypothesized that perfusion decellularization of whole intestine would result in a scaffold that allows not only for subsequent cell seeding but also for modeling of luminal-to-vascular nutrient transfer. We therefore decellularized a segment of jejunum using serial detergent perfusion to create whole-organ scaffolds with intact villous structures and perfusable vessels.

In this current study, we have repopulated the scaffold vasculature with human endothelial cells and the lumen with human iPSC-derived intestinal epithelial progenitors. In vitro biomimetic culture using arterial perfusion led to the restoration of vascular throughput and formation of continuous intestinal epithelium throughout the lumen. This successful recellularization allowed for ex vivo analysis of luminal-to-vascular nutrient transfer. We assessed in vivo engraftment of human intestinal epithelium and its absorptive capacity by adapting a previously established model of heterotopic intestinal transplantation^18^. Transplanting our regenerated intestine to the neck region of RNU rats allowed us to confirm long-term viability of our grafts and measure systemic uptake of nutrients delivered to the lumen.

## Results

### Perfusion decellularization of native intestinal scaffolds

**Fig. 1:**
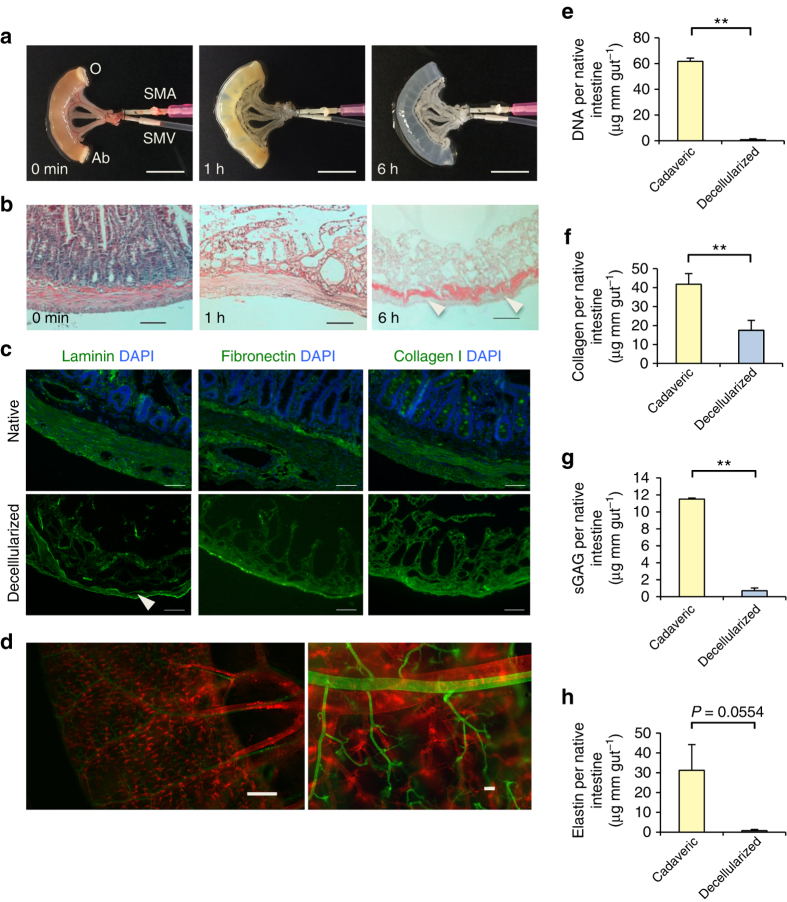
Perfusion decellularization of rat small intestinal segments. **a** Time-lapse photographs of a cadaveric rat small intestinal segment undergoing anterograde arterial perfusion decellularization. **a** Shown are a freshly isolated small intestinal segment (*left*) and the same segment after 1 h (*middle*), and 6 h (*right*) of SDS perfusion. *Scale bars*, 10 mm. **b** Representative corresponding hematoxylin and eosin stained sections of rat small intestine during perfusion decellularization. Villous projections and arterioles/venules (*arrowheads*) were preserved after decellularization (*right*). *Scale bars*, 100 μm. **c** Representative immunofluorescent stains of cadaveric and corresponding decellularized rat small intestine sections showing the preservation of extracellular matrix proteins laminin, fibronectin, and collagen I in the absence of cells. *White arrowheads* indicate the preserved vascular basement membranes. **d** A representative whole-mount image of decellularized rat small intestine perfused with green-fluorescent microspheres (0.2 µm) through the artery and red-fluorescent microspheres (0.2 µm) through the vein. *Scale bars*, 1 mm (*left*) and 100 µm (*right*). **e**–**h** Biochemical quantification of DNA, total collagen, sulfated glycosaminoglycans (sGAG), and elastin in cadaveric vs. decellularized rat small intestinal tissue showing reductions in DNA content as well as collagen, sGAG, and elastin after perfusion decellularization. The data are normalized in mass per unit length of native intestine. *Error bars*, mean ± s.d. of experimental values. *P < 0.05; **P < 0.01, Student’s *t* test. Ab, aboral end of the intestinal lumen; O, oral end of the intestinal lumen; SMA, superior mesenteric artery; SMV, superior mesenteric vein

To create a vascularized scaffold from small intestine, a 4-cm segment of proximal jejunum was isolated from Sprague-Dawley rats, with preserved perfusion from the superior mesenteric artery (SMA) through to the superior mesenteric vein (SMV). The segment represented ~4% of whole small intestine of a typical adult rat^19^. The native intestine was subjected to a series of detergent washes in order to remove all cellular components from the ECM (Fig. 1a). Histology of the acellular small intestine showed preservation of gross tissue architecture such as villus projections, arterioles and venules, and complete removal of nuclei and cellular components (Fig. 1b). Sodium dodecyl sulfate (SDS), deionized water, and Triton X-100 reduced the total DNA content per unit length of original small intestine to less than 3% (*n* = 6, 61.7 ± 2.6 vs. 8.4 · 10^−1^ ± 6.6 · 10^−1^ µg mm^−1^, mean ± s.d., *P* < 0.0001) (Fig. 1e). Immunofluorescent staining confirmed the presence of key ECM proteins such as laminin, fibronectin, and collagen I that are expressed in the native intestine^20^ in a stratified manner (Fig. 1c). In analyzing the matrix remaining after decellularization, 41% of total collagen in the native intestine was retained (*n* = 3, 41.7 ± 5.7 vs. 17.5 ± 5.3 µg mm^−1^, *P* = 0.0057) (Fig. 1f), while levels of sulfated glycolsaminoglycans and elastin were reduced to 6% (*n* = 3, 11.5 ± 1.2 · 10^−1^ vs. 7.0 · 10^−1^ ± 3.6 · 10^−1^ µg mm^−1^, *P* = 0.0001) and 2% (31.2 ± 13 vs. 7.3 · 10^−1^ ± 5.8 · 10^−1^ µg mm^−1^, *P* = 0.0554), respectively (Fig. 1g, h). To confirm the scalability of our strategy to small intestine of human scale, we successfully decellularized porcine small intestines using a similar perfusion protocol (*n* = 3, Supplementary Fig. 1a, b).

After decellularization, we confirmed preservation of perfusable channels along the hierarchical vascular bed by dye perfusion in a manner similar to that in our previously published data on perfusion of decellularized whole hearts, lungs, and kidneys^15–17^ (Supplementary Fig. 1c). Preservation of macro- and microvascular compartment was confirmed using fluorescent microspheres (Fig. 1e). Injection of 0.2 µm microspheres through SMA under physiologic pressure (80–100 mm Hg) resulted in thorough distribution into capillaries, while giving almost no flow-through into the venous system, presumably due to loss of hydrostatic pressure and collapse of venous system^21^. Preservation of the venous system was seen in subsequent injection of 0.2 µm microspheres through the SMV under 4–8 mm Hg pressure. The intact barrier between the decellularized vessel lumen and the intestinal lumen was confirmed by lack of accumulation of these fluorescent microspheres in the luminal compartment.

### Recellularization of acellular intestinal scaffolds

**Fig. 2:**
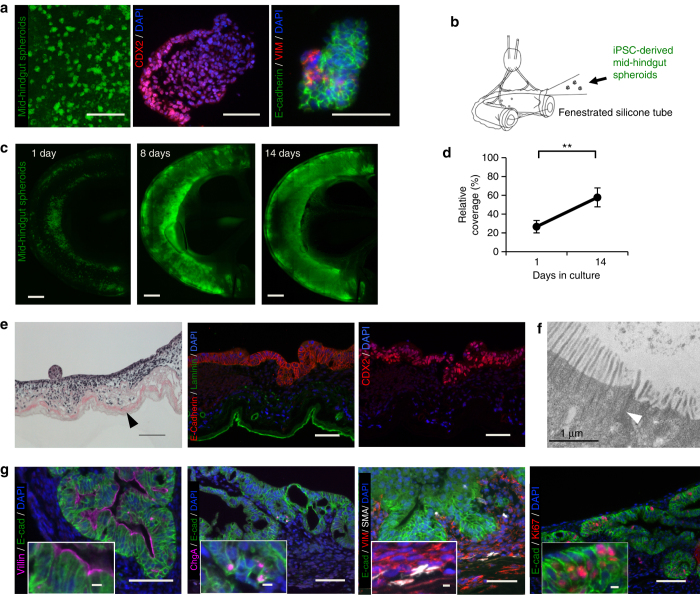
Cell seeding and whole-organ culture of decellularized rat intestine. **a** Fluorescence micrographs of GFP-positive human iPSC-derived mid-hindgut spheroids budding out from 2-dimensional culture of definitive endoderm on a tissue culture plate (*left*), a spheroid consisting of CDX2-positive cells (*middle*), a spheroid stained with E-cadherin and vimentin demonstrating cells of both epithelial and mesodermal fate (*right*). *Scale bars*, 1 mm (*left*) and 100 µm (*middle*/*right*). **b** Schematic of seeding mid-hindgut spheroids into the intestinal lumen of a rat intestinal scaffold. **c** Whole-mount fluorescence micrographs showing GFP-positive mid-hindgut spheroids proliferating to cover regions of the intestinal wall over the culture period. *Scale bars*, 2 mm. **d** Quantification of scaffold luminal coverage using visualization of GFP-positive signal at 1 day and 14 days of spheroid culture (*n* = 3). *Error bars*, mean ± s.d. of experimental values. **P < 0.01, Student’s *t* test. **e** Hematoxylin and eosin staining (*left*) and fluorescence micrographs of the intestinal epithelium engrafted on rat intestinal scaffold at day 28 of culture in vitro. E-cadherin-positive epithelial cells form a monolayer on the luminal surface of the intestine (*middle*) and express CDX2 (*right*), indicating intestinal lineage. *Scale bars*, 100 µm. **f** Transmission electron micrograph of regenerated intestinal epithelium on an intestinal scaffold. Presence of microvilli and cellular junctions (*white arrowheads*) are identified. **g** Characterization of iPSC-derived epithelium shows that villin is predominantly expressed on the apical surface of the epithelium, indicating enterocytes (*left*), a subset of cells express chromogranin A, indicating enteroendocrine cells (*middle left*), a supportive mesenchyme containing smooth muscle cells stains positive staining for vimentin and SMA (*middle right*) and a proliferative cell population is indicated by positive Ki67 staining (*right*). *Scale bars*, 100 µm; 10 µm (*insets*)

To restore absorptive capability to the intestinal scaffolds, we aimed to regenerate two key elements, the epithelium and vascular endothelium. The building blocks for our regenerated epithelium were human iPSCs modified to constitutively express green-fluorescent protein (GFP). These cells were first directed to form intestinal progenitor cells in vitro, confirmed by the presence of CDX2-positive mid-hindgut spheroids^8^, (Fig. 2a). As described in the previously published protocol, a small percentage of cells within the spheroids were vimentin^+^/E-cadherin^−^, consistent with differentiation towards a mesenchymal fate^8^ (Fig. 2a). To seed the scaffolds, a suspension of these spheroids in supportive media was injected into the lumen and the construct was then allowed to mature in vitro for 14 days. (Supplementary Fig. 2b).

Our initial attempts (*n* = 3) resulted in a successful engraftment of mid-hindgut spheroids to the scaffold and subsequent maturation into dense areas of epithelial tissue surrounding multiple disorganized neo-lumens (Supplementary Fig. 2c), similar to “organoids” described in previous reports^8, 12^. Given successful engraftment of mid-hindgut spheroids, we then attempted to distribute them more efficiently onto the luminal surface by occupying luminal space with a piece of silicone tubing (Fig. 2b). After 2 weeks of culture in a bioreactor, the area of the scaffold luminal wall covered by the GFP^+^ iPSCs increased from 27 ± 6.6% up to 58 ± 10% (*n* = 3, mean ± s.d., *P* = 0.0020), as quantified by whole-mount fluorescent microscopy (Fig. 2c, d). Notably, the addition of the silicone tube not only led to homogeneous distribution of the spheroids, but also yielded the formation of areas with a continuous epithelial monolayer, positive for E-Cadherin and CDX2, along the luminal surface (Fig. 2e), when the culture was extended up to 2 weeks or longer. The silicone tube likely acted to force the spheroids into close proximity with the supporting scaffold, rather affecting the cells themselves, as this phenomenon was not observed when the spheroids were embedded in Matrigel on plastic culture plates in proximity to pieces of silicone tube (*n* = 2, Supplementary Fig. 3a, b).

By histologic evaluation, we found that cells comprising the epithelial monolayer predominantly expressed VIL1 (Fig. 2g), characterizing them as enterocytes, which play a central role in active and passive absorption of major nutrients. These enterocytes also expressed brush-border enzymes such as Sucrase-Isomaltase and Maltase, necessary for digestive function (Supplementary Fig. 4a). Apical-basolateral polarity of the epithelial monolayer was demonstrated by apical localization of the digestive enzymes and the tight junction protein ZO-1, as well as localization of Na/K-ATPase to the basolateral side. Using transmission electron microscopy, presence of microvilli on apical surface of enterocyte-like cells was confirmed (*n* = 2, Fig. 2f, Supplementary Fig. 4b). These findings confirmed regeneration of three-dimensional epithelium on intestinal scaffolds with capability for digestive, absorptive, and barrier function.

Epithelial cell-types were also identified on the regenerated epithelium, though not the full complement of subtypes found in native intestine **(**Supplementary Fig. 5 **)**. Enteroendocrine cells, expressing Chromogranin A and enterocytes expressing villin, were present in the epithelium of scaffolds following 2 weeks of culture on a tissue culture plate **(**Fig. 2g **)**. Intestinal goblet cells were not identified by MUC2 staining and, though a high percentage of cells were weakly immunoreactive to lysozyme, fully mature individual paneth cells with strong lysozyme positivity were not convincingly characterized at this point. The presence of a proliferative cell population from which all epithelial cell-types could be replenished was confirmed by Ki-67 staining, which was positive in a fraction of the iPSC-derived cells (Fig. 2g).

The iPSC-derived spheroids seeding to the scaffolds for 2 weeks not only formed an epithelial monolayer, but also repopulated a supportive mesenchyme, as evidenced by a multicellular layer of vimentin^+^ cells underlying the basal surface of the epithelium (Fig. 2g). A subpopulation of these mesenchymal cells were vimentin^+^/smooth muscle actin (SMA)^+^, indicating the presence of intestinal subepithelial myofibroblasts (ISEMFs)^8^.

**Fig. 3:**
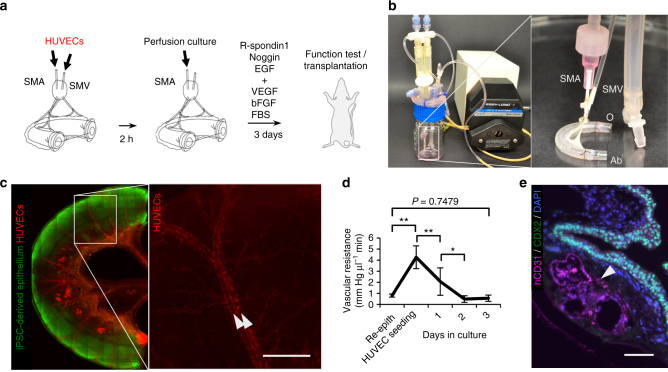
Re-endothelialization of decellularized scaffold vasculature. **a** Method of human endothelial cell culture within the scaffold vasculature, using. 2 h of static culture followed by 3 days of arterial perfusion of culture prior to ex vivo function test or transplantation experiments. **b** Custom-built bioreactor allowing for endothelial cell deliveries to scaffold artery and vein, and subsequent perfusion culture. **c** Whole-mount fluorescence micrographs of a scaffold following 14 days of iPSC-derived epithelial cell culture and subsequent seeding with RFP-labeled HUVECs for an additional day. HUVECs distributed throughout the arterial and venous vascular systems (*white arrowheads*) with minimal leakage. *Scale bar*, 1 mm. **d** Vascular resistance of re-endothelialized intestinal scaffolds (*n* = 3) under constant rate perfusion. **e** Intestinal epithelium (CDX2^+^) and endothelium (CD31^+^) engrafted on an intestinal scaffold after 17 days culture in vitro. An open capillary vessel (*white arrowhead*) is seen between an artery and a vein. *Scale bar*, 100 µm. *Error bars*, mean ± s.d. of experimental values. *P < 0.05; **P < 0.01, Student’s *t* test. Ab, aboral end; O, oral end of intestine

Following 2 weeks of static culture to achieve re-epithelialization, we sought to regenerate the endothelium necessary for perfusable vasculature by instilling a total of 1 · 10^7^ red-fluorescent protein (RFP)-labeled human umbilical venous endothelial cells (HUVEC) in a suspension with EGM-2 media through both the SMA and SMV of the intestinal constructs (Fig. 3a). After cell seeding, intestinal constructs were transitioned to culture in a whole-organ bioreactor for 3 days, which enabled constant perfusion of culture medium while maintaining a patent vascular lumen **(**Fig. 3b). Whole-mount fluorescent microscopy demonstrated distribution of RFP-labeled HUVECs within the construct, as well as further proliferation of GFP-labeled, iPSC-derived intestinal epithelium during this time (Fig. 3c). Steady decrease in vascular resistance was seen after endothelial seeding (*n* = 3, 4.3 ± 1.0 mm Hg µl^−1^ min at endothelial cell seeding, to 2.0 ± 1.2 mm Hg µl^−1^ min on day 1, *P* = 0.0075, and 4.9 · 10^−1^ ± 3.0 · 10^−1^ mm Hg µl^−1^ min on day 2, *P* = 0.0356), suggesting formation of an organized endothelium and restoration of perfusability (Fig. 3d). Immunostaining for CD31confirmed repopulation of the intestinal scaffolds’ blood vessels ranging from larger arterioles down to capillaries, in close proximity to the CDX2-positive neo-epithelium (Fig. 3e).

### Ex vivo function of acellular and regenerated intestines

**Fig. 4:**
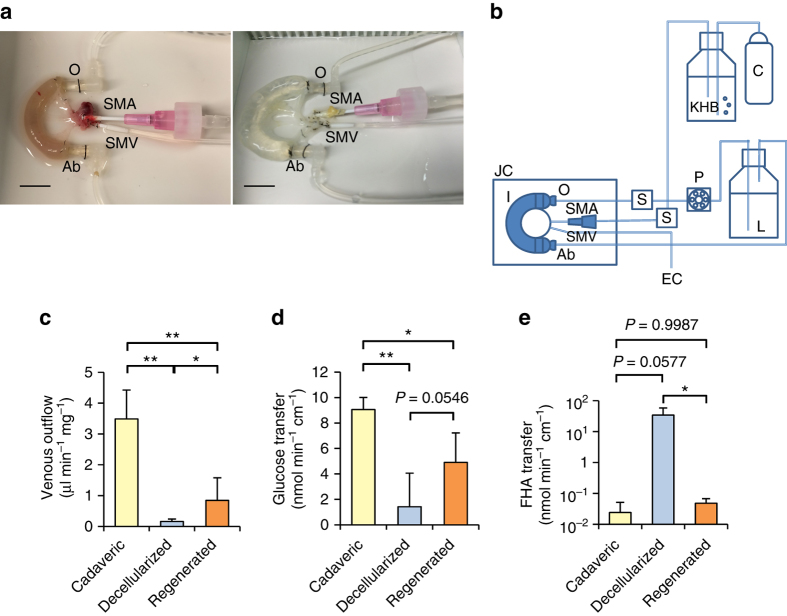
Ex vivo functional assay of bioengineered intestinal grafts. **a** Photographs of a cadaveric and a recellularized intestinal scaffold undergoing ex vivo perfusion in a thermostatic chamber. Grafts were perfused with vascular perfusate through the cannulated superior mesenteric artery (SMA), while the intestinal lumen was instilled with luminal perfusate that contained nutritional elements. Vascular effluent was collected through the cannulated superior mesenteric vein (SMV). *Scale bars*, 1 cm. **b** Diagram of the isolated perfusion circuit used for ex vivo functional analysis. Oxygenated Krebs–Heinseleit buffer (KHB) was perfused through the SMA under constant pressure of 80 mmHg, while luminal perfusate (L) was infused at a constant rate of 0.6 ml min^−1^ using a peristaltic pump (P). **c** Flow rates of vascular perfusate measured by venous effluent collection of cadaveric, decellularized, and regenerated intestines (*n* = 3), showing loss of vascular flowthrough with decellularization and partial restoration in regenerated intestines. The data were normalized by tissue mass. **d** Luminal-to-vascular glucose transfer rate of cadaveric, decellularized, and regenerated intestines (*n* = 3), showing loss of glucose absorptive function after decellularization and nearly-significant restoration in regenerated intestines. The data were normalized by length of intestine. **e** Luminal-to-vascular 6-Fluorescein-5(6)-carboxamido hexanoic acid (FHA) transfer rates of cadaveric, decellularized, and regenerated intestines (*n* = 3), showing increased FHA transfer in decellularized compared to cadaveric intestine, but a similar rate to cadaveric tissue in regenerated intestines. The data were normalized by length of intestine. *Error bars*, mean ± s.d. of experimental values. *P < 0.05; **P < 0.01, Student’s *t* test. Ab, aboral end of the intestinal lumen; C, carbogen; EC, effluent collection; JC, thermostatic jacketed chamber; O, oral end of the intestinal lumen; S, pressure sensor

We investigated the perfusability of the regenerated vasculature of our intestinal segments in an established ex vivo assay by measuring the rate of throughput from arterial inflow to venous outflow of Krebs-Heinseleit buffer under physiological conditions^22^ (Fig. 4a, b). Perfusion of decellularized small intestine produced very little venous effluent, due to the expected loss of vascular barrier function. After re-endothelialization with HUVECs, our intestine demonstrated a 5.3-fold increase in vascular throughput (*n* = 3, 1.6 · 10^−1^ ± 8.1 · 10^−2^ vs. 8.5 · 10^−1^ ± 7.2 · 10^−1^ µl min^−1^ mg^−1^, mean ± s.d., *P* = 0.0482). This regenerated vasculature demonstrated perfusability equal to 24% of that of freshly isolated cadaveric small intestine in volumetric assay (8.5 · 10^−1^ ± 7.2 · 10^−1^ vs. 3.5 ± 9.3 · 10^−1^ µl min^−1^ mg^−1^, *P* < 0.001) (Fig. 4c).

We then tested the capacity of regenerated intestinal constructs to transfer basic nutrients from a standardized luminal content across the epithelium into the venous effluent. Luminal content contained 25 mM glucose, while the perfusate infused through scaffold vasculature was devoid of glucose. The rate of glucose transfer measured in the venous effluent of fresh cadaveric small intestine was 9.1 ± 0.9 nmol min^−1^ cm^−1^ (*n* = 3, mean ± s.d.), and the rate of glucose transfer measured then decreased to 1.4 ± 2.6 nmol min^−1^ cm^−1^ after decellularization (*P* = 0.002), consistent with a baseline level of free diffusion and but loss of true absorptive epithelial function. Bioengineered intestinal grafts showed restored glucose transfer rate of 4.9 ± 2.3 nmol min^−1^ cm^−1^, a 3.5 fold increase from decellularized bowel (*P* = 0.0546). Overall, bioengineered intestine was able to transfer glucose from luminal content and transport it via effluent vasculature at a rate equal to 54% of that of fresh cadaveric bowel, suggesting partially restored vascular barrier function and epithelial absorption (Fig. 4d).

Medium-chain fatty acids (MCFAs) consumed by mouth are absorbed via passive diffusion from the gastrointestinal tract to the portal system^23^. To investigate luminal-to-vascular transfer of MCFAs in our constructs, we added 25 µM fluorescent-labeled MCFA (6-Fluorescein-5(6)-carboxamido hexanoic acid (FHA)) to the luminal perfusate. Cadaveric bowel demonstrated an FHA transfer rate of 2.4 · 10^−2^ ± 2.7 · 10^−2^ nmol min^−1^ cm^−1^. This transfer rate then increased to supraphysiologic levels after decellularization (*n* = 3, 2.4 · 10^−2^ ± 2.7 · 10^−2^ nmol min^−1^ cm^−1^ to 34 ± 24 nmol min^−1^ cm^−1^, *P* = 0.0577), reflecting loss of both epithelial and vascular barrier function in the scaffold. Following re-epithelialization and re-endothelialization, FHA transfer rate decreased and was restored to a level equivalent to that of cadaveric bowel (34 ± 24 nmol min^−1^ cm^−1^ to 4.8 · 10^−2^ ± 2.0 · 10^−2^ nmol min^−1^ cm^−1^, *P* = 0.0408) (Fig. 4e).

### Heterotopic transplantation of bioengineered intestines

**Fig. 5:**
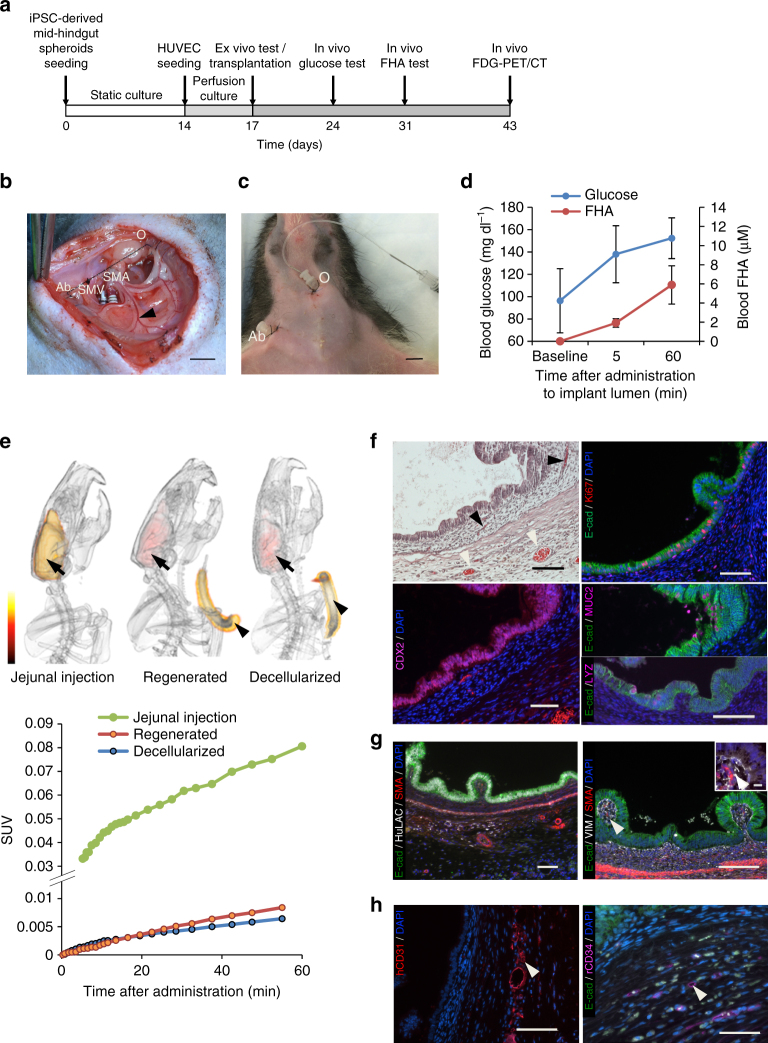
Heterotopic transplantation of bioengineered intestinal grafts and in vivo functional assays. **a** Timeline of in vivo tests after the initial seeding of human iPSC-derived spheroids. **b** Photograph of heterotopic transplantation of a regenerated intestinal construct to a subcutaneous pocket in the host rat’s right cervical area. After anastomosing the recipient’s right carotid artery to the graft SMA and the right jugular vein to the SMV, the host circulation perfused the graft. Stomas were created at oral (O) and aboral (Ab) intestinal graft ends. **c** In vivo administration of nutrient substrates via the stomas. **d** Systemic concentration of glucose (*left axis*) and 6-Fluorescein-5(6)-carboxamido hexanoic acid (FHA) (*right axis*) detected in the whole blood following in vivo administration into the lumen of the regenerated intestine (*n* = 3). **e** Positron emission tomography and computed tomography (PET/CT) image-based 3D reconstruction of the recipient rats 60 min after administration of ^18^F-fluorodeoxyglucose (FDG). The control image generated by injecting FDG directly into the jejunum of a naive rat (*n* = 1, *left*) was compared with injections into the regenerated intestinal scaffold (*n* = 1, *middle*) and decellularized scaffold (*n* = 1, *right*). *Black arrowheads* indicate intestinal scaffolds. Accumulation in the brain (*black arrows*) was quantified to generate standardized uptake value (SUV) curves (graph). **f** Characterization of regenerated intestinal grafts explanted 4 weeks after transplantation. H&E staining with capillaries (*black arrowheads*) and small-sized blood vessels (*white arrowheads*) containing red blood cells (*top left*). Ongoing proliferation is demonstrated with Ki67 staining (*top right*), maintenance of intestinal lineage verified by CDX2 expression (*bottom left*), and presence of goblet cells positive for MUC2 and paneth cells positive for lysozyme shown (*bottom right*). **g** Relationship of the human iPSC-derived epithelial and mesenchymal cells to the host-derived tissue is shown with a 10× magnification image co-stained with human lamin A + C, E-cadherin, and smooth muscle actin (*left*). Epithelial villous projections contain vimentin-positive mesenchyme and SMA-positive smooth muscle cells, consistent with intestinal subepithelial myofibroblasts (*arrowheads*) (*right*, *inset* shows higher magnification). **h** Persistence of human endothelial cells within small-sized vessels (arrowheads) is confirmed by hCD31 positivity (*left*) and rat host-derived supportive vasculature shown by endothelium positive for rat CD34 (*right*)

To demonstrate the survival and function of our vascularized bioengineered intestine in vivo, we performed experimental transplantations of the bioengineered intestine in a heterotopic position^18^. SMA and SMV of bioengineered intestine grafts were anastomosed to the right carotid artery and right jugular vein, respectively, of immunodeficient rats. Immediately after reperfusion, transplanted intestinal grafts visually appeared to have pulsatile and perfused vasculature with satisfactory hemostasis (Fig. 5b, Supplementary Fig. 6). Two weeks after transplantation, in vivo studies of glucose and fatty acid absorption were performed (Fig. 5a). After glucose solution (0.5 g ml^−1^) was administered into the intestinal lumen (Fig. 5c), elevation of whole blood glucose level from baseline of 96 ± 29 mg dl^−1^ to 138 ± 26 mg dl^−1^ at 5 min and to 152 ± 18 mg dl^−1^ at 60 min was observed (*n* = 3, Fig. 5d). With luminal input of 25 mM FHA, 1.9 ± 0.4 µM and 5.9 ± 2.0 µM FHA were detected in the whole blood at 5 min and 60 min after administering, respectively (*n* = 3, Fig. 5d).

To trace specific incorporation of luminal nutrients into host tissue, we administered glucose analog ^18^F-fluorodeoxyglucose (FDG) into the intestinal lumen and acquired images using positron emission tomography and computed tomography (PET/CT). Injection of ^18^F-FDG directly into jejunum of a naive rat (*n* = 1) as a positive control resulted in prominent accumulation in brain (Fig. 5e *left*). Bioengineered intestine 4 weeks after transplantation (*n* = 1) demonstrated accumulation of ^18^F-FDG to the brain over 60 min after luminal administration (Fig. 5e *middle*). Time activity analysis in the brain showed luminal administration into bioengineered intestine resulted in 12% SUV of that seen in the jejunal injection animal (Fig. 5e *graph*). A decellularized intestinal scaffold implanted for a similar period of time (*n* = 1) showed ^18^F-FDG equivalent to 10% SUV of the jejunal injection animal (Fig. 5e *right*).

Histological evaluation of the bioengineered intestine explanted 4 weeks after transplantation demonstrated maintained engraftment of the transplanted iPSC-derived columnar epithelium on intestinal scaffold lumen (Fig. 5f). The epithelium was confirmed to be of intestinal lineage by positive CDX2 immunoreactivity and Ki67 staining showed that cells within the columnar epithelium maintained a proliferative state. As cells formed villous projections stemming from the monolayer of epithelium, they lost their proliferative status, similar to enterocytes in native intestinal villi. In addition, MUC2-positive goblet cells and paneth cells positive for lysozyme were found in the epithelium, suggesting intestinal maturation after heterotopic transplantation (Supplementary Fig. 5). Examination of the markers vimentin and SMA showed persistence of a stratified mesenchyme, containing both ISEMFs within the villous projections and a discreet organized smooth muscle layer within the mesenchyme. Both mesenchymal and epithelial components were verified to be of human origin by staining for human lamin A + C (Fig. 5g). Presence of small sized blood vessels with human CD31 was seen (Fig. 5h *left*), although majority of blood vessels were positive for rat CD34 (Fig. 5h *right*), suggesting ingrowth of host endothelium.

Quantitative PCR was used to further compare in vitro cultured recellularized scaffolds with transplanted recellularized scaffolds of the same duration of culture (Supplementary Fig. 7). Confirming our histologic findings, transplanted bowel showed increased gene expression of most epithelial cell subtype markers, relative to in vitro cultured tissue. Relative expression of lysozyme decreased, which correlates with the immunostaining data showing that the transplanted grafts stain less strongly overall, but with more specific lysozyme-positive cells identified. Relative α-SMA expression increased, reflecting expansion of an organized layer of smooth muscle and the presence of ISEMFs. Relative EpCAM decreased, but this likely reflects expansion of the mesenchyme after transplantation. Finally, we observed increased relative expression of alkaline phosphatase, sucrose isomaltase, and GLUT2, enzymes that are characteristic of mature enterocytes and needed for absorptive function.

Examination of the ECM components in the decellularized scaffold, the scaffolds cultured for 28days in vitro, and the transplanted scaffolds for 4 weeks with immunofluorescent staining showed the core ECM components collagen I, collagen IV, fibronectin, and laminin surrounding the iPSC-derived cells (Supplementary Fig. 8). GAGs, elastin, and collagen, all of which were diminished with the decellularization process, were examined with Alcian Blue, Verhoeff-Van Gieson, and Masson’s Trichrome stains respectively. These ECM components appeared partially restored in the in vitro cultured scaffolds and were visualized to an even greater degree in the scaffolds after transplantation (Supplementary Fig. 9). Immunostaining with a human species-specific collagen IV antibody showed no reactivity in the rat-derived decellularized scaffolds, but areas of positive staining surrounding the iPSC-derived cells in both the in vitro and transplanted recellularized tissue (Supplementary Fig. 10), implying that cells deposit ECM onto the constructs as they engraft.

## Discussion

An implantable bioengineered intestinal graft generated from patient-derived cells would provide an alternative treatment strategy for patients suffering from SBS. In the present study, we found that detergent-based perfusion decellularization of whole intestinal segments provides an acellular scaffold comprised of native ECM with preserved vascular and villous ultrastructure. We showed scalability of this technique from rat to porcine intestine. On the basis of this platform, we describe a new approach to bioengineer intestinal grafts and report three milestones: the repopulation of endothelial and epithelial compartments using human-derived endothelial cells and iPSC-derived intestinal epithelial progenitors; absorptive function of the perfusable graft in ex vivo testing; and survival of the bioengineered human intestinal tissue with preservation of basic function of the grafts following long-term heterotopic transplantation in vivo.

Decellularized scaffolds show complete loss of cell-mediated functions such as small molecule barrier, digestion, and nutrient absorption. To engineer a viable graft with clinically relevant function, we aimed to restore ability to transfer nutrients via forming sustainable epithelium with absorptive function and perfusable vasculature with viable endothelium. We differentiated human iPSCs into intestinal lineage cells in vitro by applying an established protocol^8, 24^. We took advantage of the phenomenon that human iPSC-derived endoderm forms free-floating spheroids during in vitro differentiation into mid-hind gut. These spheroids primarily contained CDX2-positive cells, which enabled naturally selective collection and seeding of intestinal epithelial progenitors to regenerate the epithelial component of the intestine. The ability of the cells comprising these spheroids to form an intestinal epithelium has been previously shown^8, 12^. In addition to the original method of embedding in Matrigel, attempts to differentiate and culture on transwell inserts^25^ and dissecting organoids in halves and placing on scaffolds^26^ have previously been made. However, a method to drive spheroids to form single epithelialized lumen on a vascularized construct has not been reported to date.

In a set of pilot experiments, we determined the optimal time point to transfer cells to the three dimensional environment of intestinal matrix. Cells further allowed to mature in vitro past 11 days formed the expected multiple independent epithelial lumens similar to intestinal organoids when transferred onto intestinal matrix (Supplementary Fig. 2e). Application of a silicone surface opposed to the intestinal basement membrane, led to establishment of epithelial polarity, and regions of continuous epithelial coverage on the lumen of the scaffold. Epithelial cell adherence and monolayer formation may be explained by physical aposition, growth factor gradient, hydrophobicity of the silicone surface, or a combination of these factors.

Over the in vitro culture period, we observed transformation of progenitors on the scaffold into a monolayer of epithelium, which contained a subset of the intestine-specific cell subtypes and had apical-basolateral polarity. We did not detect differentiation of goblet cells or paneth cells, possibly due to insufficient in vitro culture time on the scaffold or differences in growth factor availability on the scaffold in comparison to traditional 3D culture within Matrigel. However, after our bioengineered epithelium was placed in the in vivo culture environment provided by 4 weeks of transplantation, we noted the appearance of MUC2-positive cells indicative of goblet cells and specific lysozyme-positive cells consistent with paneth cells. Additionally, the transplanted tissue demonstrated expansion of the mesenchyme, particularly the SMA^+^ smooth muscle cells which formed a discreet layer.

Human enterocytes are known to turn over every 3.5 days, so any sustainable intestinal graft must contain a proliferative cell population from which to renew the epithelium^27^. In both the in vitro cultured and the transplanted bioengineered intestine, a population of the iPSC-derived epithelial cells maintained a proliferative state. Overall, the architecture of our regenerated intestinal grafts, with a monolayer of epithelium and small budding villous projections of non-proliferative cells, appeared less mature than that of native intestine, which contains longer villous projections with a crypt-niche at the base. However, the increase in scaffold area coverage over the 28 day in vitro culture period and the persistence of a mature epithelium after a 28 day period of transplantation both point to the presence of a durable renewable cell population that will support the graft long-term.

Developing the scaffold and culture conditions to establish a contiguous layer of epithelium on acellular matrix was an important step. However, absorptive function at a scale enabling nutritional support requires a perfusable vascular bed supporting epithelial function. We therefore aimed to re-endothelialize intestinal scaffolds via the intact native vascular channels. After endothelial seeding and perfusion culture in vitro, intestine constructs demonstrated improved vascular flow-through. Histologic analysis confirmed intact endothelium with patent vascular lumen close to regenerated intestinal epithelium, representing the minimal requirements for a nutrient intake unit. The intact vascular architecture of bioengineered intestines provided the opportunity to test intestine-to-vascular nutrient transfer both ex vivo and in vivo, that would not have been possible in traditional two-dimensional culture.

To test bioengineered intestinal graft function in vitro, we adapted an isolated bowel model. Comparison of vascular throughput in decellularized versus re-endothelialized constructs demonstrated a significant improvement in perfusability, indicating coverage of the vascular lumen with endothelial cells sufficient to generate barrier function and a patent vascular lumen. Regeneration of vascular perfusion to our constructs allowed for testing of nutrient transfer from lumen to the vascular space.

We first measured glucose absorption to test the ability of the constructs to transfer simple carbohydrates. Glucose administered to the lumen of continuously perfused constructs decellularized constructs resulted in a low level of absorption consistent with free diffusion. Re-epithelialized constructs, however, demonstrated increased glucose transfer that neared significance in comparison with decellularized scaffolds. Because our lumen was not uniformly covered with epithelium, the glucose transfer by our constructs represents a combination of free diffusion from the acellular areas of the scaffold, which lack the tight junctions reconstituted by re-epithelialization, and cell-mediated uptake where cell engraftment has taken place. The absorptive function of our neo-epithelium is still below the level of that of cadaveric bowel, which is expected due to the partial coverage of the scaffold and the reduced surface area provided by the monolayer with small villous projections. However, the clear improvement of recellularized constructs over decellularized scaffolds points to a component of active cellular absorption. Furthermore, the confirmation of digestive enzymes on our iPSC-derived enterocytes suggests capacity to breakdown complex sugars, whose components could then be transferred to the vascular compartment.

Medium-chain fatty acids are directly absorbed from the intestinal lumen and transported straight to the portal blood stream. Our ex vivo studies of decellularized tissue showed a high rate of transfer to the venous compartment likely because these scaffolds completely lacked epithelial and endothelial barrier functions. A more physiologic rate of MCFA transfer can be estimated by that of the cadaveric bowel. Analysis of recellularized bowel found that the transfer rate of MCFAs was appropriately less than that of decellularized bowel and not significantly different from fresh cadaveric bowel. The regenerated intestine therefore not only permits absorption of MCFAs, an essential nutrient, but also has relatively reconstituted barrier function when compared to decellularized tissue. Generating this composite intestinal tissue with absorptive epithelium and the ability to transport nutrients in the vascular compartment makes our model a first step in creating a clinically relevant graft.

Heterotopic intestinal transplantation allowed for analysis of our bioengineered intestinal constructs in a manner that was minimally invasive to the host animals. Application of this model was made possible by utilizing a vascular pedicle, which is unique to our grafts. We identified human-origin endothelial cells 4 weeks after transplantation, although majority of blood vessels were identified as host origin. Over the long term, local ingrowth of microvasculature likely supported the survival and function of grafts.

We not only observed the survival of the seeded human-derived cells, but also found that epithelial monolayer with columnar architecture was preserved after transplantation. In addition, our intestinal epithelium continued to mature in vivo, evidenced by the presence of all epithelial subtypes, development of regions with villous architecture, and expansion of the mesenchyme. This maturation of iPSC-derived epithelium was likely due to a combination of influences, including circulating growth factors from the recipient animal, similar to that seen when iPSC-derived intestinal organoids were implanted under the kidney subcapsule in previous studies^12^. Additionally, in normal gastrointestinal development, regional signals from the mesoderm are essential for intestinal patterning and development, so the changes in the morphology and cell characteristics of our neo-epithelium after transplantation may have been due to the close association with supportive mesenchyme^28^. Finally, it is known that ECM proteins play key roles in guiding intestinal morphogenesis and in directing the proliferation, adhesion, differentiation and survival of epithelial cells by acting as ligands for cell receptors^20, 29^. Our observation that core ECM components, which are diminished in the decellularization process, are partially restored after recellularization in vitro and reconstituted to a greater degree in vivo, points to a role for the ECM in influencing our tissue maturation.

The survival of our bioengineered intestinal grafts after 4 weeks of transplantation allowed for basic functional analysis, including the administration of glucose and FHA, which were both detected in whole blood after luminal administration. Administration of ^18^F -FDG to the recellularized graft showed accumulation in the brain, but at a lower level than that of the native jejunum. However, because the length of our graft equated to ~4% of that of adult rat intestine, and we expected a lower degree of FDG absorption compared with in situ jejunal injection. When compared to the decellularized scaffold, the uptake from the recellularized graft appeared slightly increased but was ultimately similar in scope, reflecting the fact that some glucose does likely leak from the acellular portions of the scaffold. However, there is likely a cellular contribution as well, as evidenced in the ex vivo studies, and a decellularized graft on its own has no clinical application. Further work to improve epithelial surface area by expanding scaffold coverage and developing a more mature crypt-villous architecture would increase the role of the enterocytes in nutrient absorption.

In the field of tissue-engineering, there is currently a gap between cellular regeneration and organ regeneration. Our strategy of growing progenitor cells on vascularized scaffolds has the potential to bridge this gap, and represents a significant advance in the strategy of organ regeneration using patient-iPSC-derived tissues and whole-organ scaffolds. Our bioengineered intestine at present consists of two sources of cells: endothelial cells and epithelial cells. Further development of this model will entail the addition of a functional enteric nervous system^30^ to aid in secretory function and motility.

Although not yet sufficiently mature to totally replace native intestine, the strategy of adding our bioengineered constructs as a small segments of additional absorptive surface area could become a treatment option for nutrient intake in SBS in the future.

In summary, cadaveric small intestine in a rat model can be decellularized, repopulated with human endothelial and iPSC-derived intestinal epithelial progenitors, matured to functional intestinal constructs in vitro, transplanted, and re-vascularized to provide basic “absorptive” function in vivo. Translation of this technology beyond proof of principle will require the optimization of cell-seeding protocols to human-sized scaffolds, an upscaling and improvement of human iPSC differentiation, and maturation of vascular engineering.

## Methods

### Perfusion decellularization of intestines

All animal experiments were approved by the Massachusetts General Hospital Institutional Animal Care and Use Committee (protocol 2014N000308) and were performed in compliance with the Animal Welfare Act. We isolated 156 rat small intestines for perfusion decellularization. We anesthetized male, 8- to 12-week-old Sprague-Dawley rats (Charles River Laboratories) using inhaled isoflurane (Baxter). After median laparotomy and systemic heparinization, SMA and SMV were dissected and divided, leaving the second to fourth mesenteric vessels intact. All small vessels branching from SMA or SMV were ligated and divided. Extra care was taken to ligate all lymphatic vessels. After euthanasia by aortic exsanguination, the SMA was cannulated with a 20-gauge I.V. catheter (Smiths Medical) to allow anterograde arterial perfusion of ice-cold heparinized phosphate-buffered saline (PBS) at 90 mm Hg arterial pressure for 1 min to remove residual blood. SMV was also cannulated with a 20-gauge catheter. Jejunal wall was transected at the first and fifth mesenteric vessels, and marginal vessel stumps were ligated. Intestinal lumen was flushed with 20 ml of ice-cold PBS. We then administered decellularization solutions through the SMA at 90 mm Hg of constant pressure in the following order: 200 ml of 0.1% SDS (Thermo Fisher Scientific) in deionized water, 200 ml of deionized water, 200 ml of 1% Triton X-100 (Sigma-Aldrich) in deionized water. Resulting scaffolds were sterilized by perfusing 100 ml of 1% hydrogen peroxide and 0.08% peracetic acid (Spor-Klenz, STERIS), and were washed with 2 l of PBS to remove residual detergent and cellular debris. The scaffold was stored in PBS containing 100 units ml^−1^ of penicillin, 100 µg ml^−1^ of streptomycin, and 0.25 µg ml^−1^ of amphotericin B (Antibiotic-Antimycotic, Thermo Fisher Scientific) at 4 °C.

### Generation and maintenance of iPSCs

Normal human peripheral blood was obtained from donors with informed consent (Boston University Medical Center Institutional Review Board protocol H-32506). Human iPSC clone BU6 was generated from the peripheral blood. In accordance with published protocols, peripheral blood mononuclear cells were collected and expanded for 9 days^31–33^ in medium containing ascorbic acid, SCF, IGF-1, IL-3 and EPO before being transduced with a constitutive single excisable polycistronic lentiviral Stem Cell Cassette (STEMCCA) lentivirus encoding the Yamanaka factors. Cells were then plated onto MEFs, and at 2 weeks after infection, clones from ESC-like colonies were expanded and tested for the expression of the pluripotency markers SSEA-4, Tra-1-60, and Tra-1-81^31^. BU6 was modified to constitutively express GFP driven by CAG promoter, by introducing a CAG-GFP construct into AAVS1 locus by ZFN-mediated homologous recombination^34^. The ZFN plasmid and the GFP donor was a kind gift of Dr. Paul Gadue from The Children’s Hospital of Philadelphia to G.M. BU6 iPSC were grown in feeder-free conditions in six-well Nunclon surface plates coated with hESC-qualified Matrigel (Corning) and were maintained in mTeSR1 medium (STEMCELL Technologies) containing Primocin (100 µg ml^−1^, InvivoGen) in a 5% CO_2_ incubator at 37 °C. Cells were passaged onto a new plate every 6-7 d using ReLeSR (STEMCELL Technologies).

### Human mid-hindgut spheroid and intestinal organoid culture

On the basis of the established methods, human intestinal organoids were generated and maintained^8, 24^. Specifically, for induction of definitive endoderm (DE), human iPSCs were passaged with ReLeSR and plated at a density of 100,000 cells per well in a Matrigel-coated, Nunclon surface 24-well plate. At the confluency of 80-95%, cells were treated with Activin A (100 ng ml^−1^, R&D Systems) for 3 d with escalating supplementation of dFBS (0 to 2%). DE was then treated with hindgut induction medium (RPMI 1640, 2 mM L-glutamine, 2% defined FBS (Hyclone), and Primocin (100 µg ml^−1^)) for 4 d with FGF4 (500 ng ml^−1^,R&D Systems) and CHIR99021 (3 µM, Stemgent) to induce formation of mid-hindgut spheroids. Spheroids were collected and were either plated in Matrigel or seeded onto intestinal scaffolds described below. Spheroids were grown in intestinal growth medium (Advanced DMEM/F-12, 2% B27 Serum-Free Supplement (Thermo Fisher Scientific), 15 mM HEPES, 2 mM L-glutamine, and Primocin (100 µg ml^−1^)) supplemented with recombinant human EGF (100 ng ml^−1^, R&D Systems), Noggin (100 ng ml^−1^, R&D Systems), and R-spondin 1 (500 ng ml^−1^, R&D Systems) to generate human intestinal epithelium. Medium was changed twice weekly. Cells were confirmed to be negative for mycoplasma contamination.

### Spheroid seeding

Scaffolds were coated by injecting 80 µg of Matrigel diluted in 400 µl of DMEM/F-12 with 15 mM HEPES (STEMCELL Technologies) into the lumen followed by 10-min incubation at 37 °C. Scaffolds were then rinsed in ice-cold PBS. A platinum-cured silicone tubing (3.175 mm OD, SILASTIC, Dow Corning) was cut into 45 mm-length pieces. Ten 0.1 mm diameter holes were fenestrated in the middle area of each piece to allow fluid diffusion. One sterilized piece of the silicone tubing was inserted into the intestinal lumen of a scaffold, and was kept in “U”-shape using a 6-0 silk suture to avoid tensioning of the mesenteric vessels. Spheroids were collected from 12 to 24 wells of a 24-well tissue culture plate that underwent mid-hindgut differentiation, pelleted, and resuspended in 100 µl of intestinal growth medium. Spheroid suspension was injected into the space inside the intestinal lumen of the scaffold but outside the silicone tubing. Suture ties were applied at both ends of the scaffold lumen over the silicone tubing to contain the injected spheroid suspension (Fig. 3b).

### HUVEC preparation and seeding

We purchased GFP- or RFP-labeled HUVECs (CellPlayer HUVEC CytoLight Green/Red, Essen Bioscience) and maintained them in 0.1% gelatin (Sigma-Aldrich)-coated flasks with complete EGM-2 (Lonza) supplemented with 1% penicillin-streptomycin (Gibco). At the time of seeding, cells were trypsinized, centrifuged, resuspended in 10 ml of EGM-2, filtered through a 70 µm strainer, counted and subsequently seeded into decellularized intestines as described below. Prior to endothelial cell seeding, sterile PBS was manually injected through arterial or venous cannulae of intestinal scaffolds to identify potential vascular leakage. Only scaffolds with no major vascular leakage were used for subsequent experiments. We collected and diluted 3 to 11 million HUVECs in 4 ml EGM2 and seeded these onto the acellular intestinal scaffold through the venous cannula at a constant pressure of 10 mm Hg. Cells were allowed to attach 2 h, after which perfusion culture from vein was initiated. After 24 h, we prepared the same number of HUVECs in 4 mL EGM2 and additionally seeded them through the arterial cannula at a constant pressure of 90 mm Hg. Cells were allowed to attach 2 h, after which perfusion culture was switched from artery. At this point, the culture medium was changed into a 1:1 mixture of Advanced DMEM/F-12 and Medium 199 (Gibco) supplemented with full strength of factors contained in the intestinal growth medium and the vascular stabilization medium previously described^21^: 2% B27 Serum-Free Supplement, 15 mM HEPES, 2 mM L-glutamine, 1% Insulin-Transferrin-Selenium (Gibco), ascorbic acid (50 µg ml^−1^, STEMCELL Technologies), 10 µM forskolin (Cayman Chemical), 110 nM hydrocortisone (Sigma-Aldrich), recombinant human VEGF (20 ng ml^−1^, PeproTech), bFGF (5 ng ml^−1^, PeproTech), EGF (100 ng ml^−1^), Noggin (100 ng ml^−1^), R-spondin 1 (500 ng ml^−1^), and Primocin (100 µg ml^−1^).

### Bioreactor design and whole-organ culture

We designed and custom built the intestine bioreactor as a closed system that could be autoclaved after cleaning and assembly, needing only to be opened at the time of organ placement or whole-mount imaging. Perfusion media and cell suspensions were infused through swabbable injection ports (Quosina) to minimize the risk of contamination. Media were allowed to equilibrate with 5% CO_2_ and 95% room air by flowing through a silicone tube oxygenator (SILASTIC, Dow Corning) followed by a bubble trap before reaching the cannulated SMA and SMV at a constant flow rate of 1.2 ml per hour. Whole-mount images were acquired without losing sterility by transferring the intestine into a 6-well tissue culture plate.

### Isolated intestine experiments

Absorptive capacity of fresh cadaveric, decellularized, and regenerated intestines were assessed using the model of oxygen carrier-free, isolated perfused rat small intestine^22^. A custom-made perfusion chamber consisted of moisturized chamber with heating block and tubing connectors with bubble trap and pressure transducers (Fig. 3b).

Fresh cadaveric intestines were collected identically as described above for perfusion decellularization, with the exception that the SMA was flushed with 4 °C Belzer UW Cold Storage Solution (Bridge to Life) at isolation^35^. After securing onto the chamber, Storage Solution was washed away with single flush of PBS. In regenerated intestine group, the silicone tubing used at spheroid seeding was removed from the lumen prior to securing onto the chamber.

Vascular perfusate was prepared with substitution of mannitol for glucose to allow for analysis of glucose transfer. Specifically, vascular perfusate was a modified Krebs-Henseleit solution containing 2 mM lactobionatic acid, 48.5 mM mannitol, 0.8 mM L-glutamine, 6.7·10^−5^ mg l^−1^ norepinephrine hydrochloride, 12.6 mM HEPES, and 3%(w/v) bovine serum albumin (MP Biomedicals). The buffer was maintained at 37 °C while oxygenated with carbogen gas (95% O_2_, 5% CO_2_), and pH was equilibrated at 7.4. For perfusion, we applied a constant gravity pressure of 90 mm Hg through arterial cannula.

Luminal perfusate contained 114 mM NaCl, 5 mM KCl, 26 mM NaHCO_3_, 30 mM lactose, 25 mM (4.5 g l^−1^) glucose, 10 mM mannitol, and 0.8 mM L-glutamine. After priming the intestinal lumen by manual infusion, luminal perfusate was infused at constant flow rate of 0.6 ml min^−1^. For the fatty acid absorption experiment, 25 µM 6-Fluorescein-5(6)-carboxamido hexanoic acid (FHA) was added in the luminal perfusate.

Venous effluent was collected through a small tubing (1.65 mm OD, SILASTIC, Dow Corning) into a microcentrifuge tube placed 4 cm below the venous cannula. The priming volume of 200 µl was excluded from the first effluent collection. Total volume of effluent samples was recorded and the solution then deproteinized using Amicon Ultra 0.5 ml centrifugal filters (10 kDa). All reagents were from Sigma-Aldrich unless otherwise noted.

### Heterotopic transplantation of intestine

The rat model of cervical transplantation of small intestine has been previously described^18^. We employed a cuff technique^36^ to perform end-to-end anastomoses of graft SMA and SMV to recipient right carotid artery (RCA) and right jugular vein (RJV), respectively. Intestine grafts were prepared for transplantation by placing a cuff created from a 20-gauge I.V. catheter (Smiths Medical) in place of the graft SMV cannula (Supplementary Fig. 5). As recipients, 12- to 16-week-old NIHRNU rats (300-500 g, Harlan/Envigo) were used. After skin incision and systemic heparinization (300 unit kg^−1^), RCA was distally ligated and transected. The proximal stump was tunneled behind the right sternocleidomastoid muscle to bring closer to the RJV. Proximal clamp was placed on the RCA and a 20-gauge cuff was placed on the stump. Graft SMA cannula was removed, and cuffed host RCA was inserted and secured with a 6-0 silk ligation (Fine Science Tools). Host RJV was ligated distally, and clamped proximally. Cuffed graft SMV was inserted and secured. After topical rewarming of the graft with 37 °C saline, the recipient artery and vein were unclamped, and patent anastomoses were confirmed. The graft was placed in the space created under the skin. Skin was closed after stomas were created at both ends of intestinal lumen. Two doses of buprenorphine (10 µg kg^−1^) were given subcutaneously at an interval of 12 h post-operation. Infected or deceased animals were excluded from the study.

### In vivo glucose and fatty acid absorption study

Six to seven days after transplant surgery, glucose absorption study was performed. Recipient rats were fasted 12 h prior to the experiment, underwent 5% inhaled isoflurane induction, and were maintained with 2-3% inhaled isoflurane. Rats were placed supine on a heating pad. The piece of fenestrated silicone tube that was kept in the lumen of regenerated intestine implant was flushed once with PBS. A baseline blood glucose measurement was performed from a tail puncture using CONTOUR NEXT Meter (Ascensia Diabetes Care US). After the dead space of the fenestrated silicone tube was filled via the cannulated oral end of the construct, the aboral end was plugged and an additional 100 µl of glucose solution (0.5 g ml^−1^) injected, forcing the solution to exit the tube and contact the re-epithelialized lumen of the implant. Blood glucose was measured at 5 and 60 min after luminal injection.

Two weeks after transplant surgery, FHA absorption study was performed as a terminal experiment. Under general anesthesia, a baseline blood sample (800 µl) was collected from the femoral vein. 100 µl of FHA solution (25 mM) was administered into the lumen of the implant. 400 µl of blood was collected at 5 and 60 min. Animals were euthanized after the last blood sampling. Serum was isolated from each sample, and was deproteinized for subsequent fluorescence-based quantification.

### FDG-PET/CT imaging

Subject rat was fasted overnight before imaging, and was anesthetized using inhaled isoflurane. MicroPET/CT imaging was performed using a small animal scanner (Inveon, Siemens Medical Solutions, Inc., Malvern, PA). CT imaging preceded PET imaging, with an acquisition of 360 cone beam projections using an 80 kVp, 500 µA X-ray tube. ^18^F-FDG solution (100-150 µCi in 200 µl, PETNET Solutions, Woburn, MA) was either injected into the fenestrated silicone tube kept inside the lumen of the implanted intestine, or directly injected into the jejunum under laparotomy. Dynamic PET scan was performed over 60 min. CT projections were reconstructed using a modified Feldkamp cone beam reconstruction algorithm (COBRA, Exxim Inc. Pleasanton, CA) into 3-dimensional volumes containing 512 × 512 × 768 voxels with dimensions of 0.11 × 0.11 × 0.11 mm. PET images were reconstructed using an 3D ordered subset expectation maximization maximum a posteriori (3DOSEM/MAP) algorithm with 2 OSEM iteration and 20 MAP iterations into 128 × 128 × 159 image matrix with a voxel size was 0.797 × 0.861 × 0.861 mm. Manual regions of interest were drawn and SUV’s and time activity curves were obtained using the Inveon Research Workplace (Siemens Medical Solutions Inc., Malvern, PA). Image visualizations and renderings were performed in Osirix (Pixmeo SARL, Geneva, Switzerland) and Amira (FEI, Hillsboro, OR) software packages.

### Glucose and fatty acid quantification

Glucose concentration in the deproteinized effluent or plasma samples was quantified using Glucose Assay Kit (Abcam), as per the manufacturer’s instructions. 10 µl of diluted samples were added to make a total of 100 µl of each reaction mixture. Black half-area assay plates (Corning) were used. After 30 min of incubation at 37 °C, fluorescence of the probe was read using SpectraMax Microplate Reader (Molecular Devices) at 535 nm (ex)/587 nm (em). FHA was quantified using SpectraMax Microplate Reader at 481 nm (ex)/520 nm (em). Concentrations were determined on the basis of a standard curve generated in parallel, and transfer rates were normalized to tissue wet weight.

### Histology and immunofluorescence

Tissue specimens were fixed overnight in 4% paraformaldehyde in PBS, embedded in HistoGel (Richard-Allan Scientific), dehydrated, paraffinized, and embedded in paraffin blocks. For control tissues, a formalin-fixed, paraffin embedded human small intestine normal tissue block was purchased (amsbio). Five-micron sections were generally used for histological analysis. Sections were deparaffinized, rehydrated, and stained with either Hematoxylin QS (Vector Laboratories) and eosin Y (Thermo Fisher Scientific), Alcian Blue (American Mastertech), Verhoeff-Van Gieson (American Mastertech), or Masson’s Trichrome (American Mastertech) dyes,, and mounted with Permount (Thermo Fisher Scientific) after dehydration. For immunofluorescence staining, sections were deparaffinized, rehydrated, subjected to heat-induced antigen retrieval, permeabilized with 0.1% Triton X-100 in PBS, washed three times in PBS and blocked with 1% BSA in PBS. Sections were incubated with primary antibodies overnight at 4 °C, washed three times in PBS, and then incubated with secondary antibodies for 30 min at room temperature. A full list of primary and secondary antibodies with respective dilutions has been provided (Supplementary Tables 1 and 2 **)**. Sections were then washed and mounted using DAPI Fluoromount-G (SouthernBiotech). All stained sections were imaged using a Nikon Eclipse TE200 microscope and imaging software NIS-Elements (Nikon Instruments, Melville, NY).

### Transmission electron microscopy

Tissue specimens were fixed in 2% paraformaldehyde and 2.5% glutaraldehyde in 0.1 M sodium phosphate buffer (pH 7.4, Electron Microscopy Sciences, Hatfield, PA), overnight at 4 °C, then rinsed several times in 0.1 M cacodylate buffer. Specimens were post-fixed in 1.0% osmium tetroxide in cacodylate buffer for one hour at room temperature, rinsed several times in buffer, dehydrated through a graded series of ethanols to 100%, dehydrated briefly in 100% propylene oxide, and pre-infiltrated in a 1:1 mix of Eponate resin (Ted Pella, Redding, CA) and propylene oxide overnight on a gentle rotator. The following day, specimens were infiltrated with fresh Eponate resin for several hours, embedded in flat molds with fresh Eponate and allowed to polymerize overnight at 60 °C. Thin (70 nm) sections were cut using a Leica EM UC7 ultramicrotome, collected onto formvar-coated grids, stained with uranyl acetate and Reynold’s lead citrate and examined in a JEOL JEM 1011 transmission electron microscope at 80 kV. Images were collected using an AMT digital imaging system (Advanced Microscopy Techniques, Danvers, MA).

### mRNA Isolation and quantitative PCR

RNA was isolated from tissue that had been fixed in 4% paraformaldehyde in PBS overnight and embedded in paraffin blocks. For both in vitro cultured and transplanted bowel, three unique pieces of tissue each were compared, with samples taken a minimum of 0.5 cm in distance apart. Each sample consisted of four 15 μm sections that were deparaffinized with xylene. mRNA was isolated using the RNeasy FFPE Kit (Qiagen) and transcribed to cDNA (Invitrogen SuperScript III). Gene expression was analyzed using Taqman probes and the OneStep Plus system (Applied Biosystems). Each sample was analyzed in 2 replicate qPCR reactions, with the Ct value of each replicate averaged and treated as an *n* = 1 unique biologic sample. Gene expression for each sample was normalized to human β-Actin (ACTA1) expression (ΔCt) and relative to human jejunum control samples (DDCt), with fold change calculated by 2-ΔΔCt^37^.

### Quantification of intestinal luminal coverage

For quantification of intestinal luminal coverage in regenerated intestines, fluorescence images were taken using Nikon Eclipse TE200 microscope to visualize cells that originated from GFP-positive human iPSCs. Images at 4× magnification were stitched to generate whole-mount regenerated intestine images. Images were converted to binary images using ImageJ (NIH). Pixel numbers of processed images were counted using ImageJ, which indicated the areas of either GFP-positive signals or presence of intestinal scaffold in the entire field. Intestinal luminal coverage of regenerated intestines was defined as GFP-positive area normalized to area of intestinal scaffold.

### SDS and DNA and ECM component quantification

SDS was quantified using SDS Detection Kit (G-Biosciences) in the method previously described^38^. Briefly, lyophilized tissues were digested in Collagenase buffer (Sigma) for 48 h at 37 °C with gentle rotation. Digest supernatants (1 µl) containing any residual SDS were then added to 2 ml Blue Dye and 1 ml Dye Extraction Buffer of the Kit as per manufacturer’s instruction. Absorbance was measured at 600 nm. DNA was quantified following genomic DNA extraction and purification protocol^39^. Briefly, fresh tissue samples were digested in buffer containing 400 mM NaCl, 1% SDS, 20 mM Tris-Cl (pH 8.0), and 5 mM EDTA (pH 8.0) with Proteinase K (200 µg ml^−1^, Sigma) and RNAse A (10 µg ml^−1^, Sigma-Aldrich) for 2 h at 55 °C with gentle rotation. DNA was concentrated from the digests by phenol-chloroform extraction and ethanol precipitation. Quantity and purity of DNA recovered in TE buffer were assessed base on absorbance at 260, 280, and 320 nm (NanoDrop 1000, Thermo Fisher Scientific). Soluble collagen was quantified using the Sircol Assay (Biocolor), as per the manufacturer’s instructions. Lyophilized tissue samples were first subjected to acid-pepsin collagen extraction overnight at 4 °C and then to overnight isolation and concentration. Assay was then performed as instructed. Sulfated glycosaminoglycans (sGAG) were quantified using the Blyscan Assay (Biocolor). Before measurement, sGAG were extracted using a papain extraction reagent (Sigma- Aldrich) and heated for 3 h at 65 °C. Assay was then performed as instructed. Elastin was quantified using the Fastin Assay (Biocolor) as instructed. All concentrations were determined on the basis of a standard curve generated in parallel, and values were normalized to original tissue wet weight.

### Statistical analysis

Sample sizes were chosen to enable detection of physiologically relevant differences between groups, and calculated based on the historical data. Animal experiments were performed to have biological triplicates for each group, except for PET/CT imaging (*n* = 1 per experimental arm). No randomization was performed. Investigators were not blinded. Statistical analyses were performed by Student’s *t* tests (two-tail comparisons) and statistically significant differences were defined as *P* < 0.05. Values in graphs were presented as means with s.d. Microsoft Excel 2016 for Mac (Microsoft Corporation) and JMP 13 (SAS Institute) were used for the data management, statistical analyses and graph generation.

### Data availability

The authors declare that all the data supporting the findings of this study are available within the article and its Supplementary Information Files, or from the corresponding author (H.C.O.) upon reasonable request.

## Electronic supplementary material

Supplementary Information
