# Supplementary material for: Bioengineering of functional human induced pluripotent stem cell-derived intestinal grafts
Source: Nat Commun. 2017 Oct 10;8:765. doi: 10.1038/s41467-017-00779-y (PMC5635127; doi:10.1038/s41467-017-00779-y)
Supplement: Supplementary file 1 — Supplementary Information [file 41467_2017_779_MOESM1_ESM.pdf]

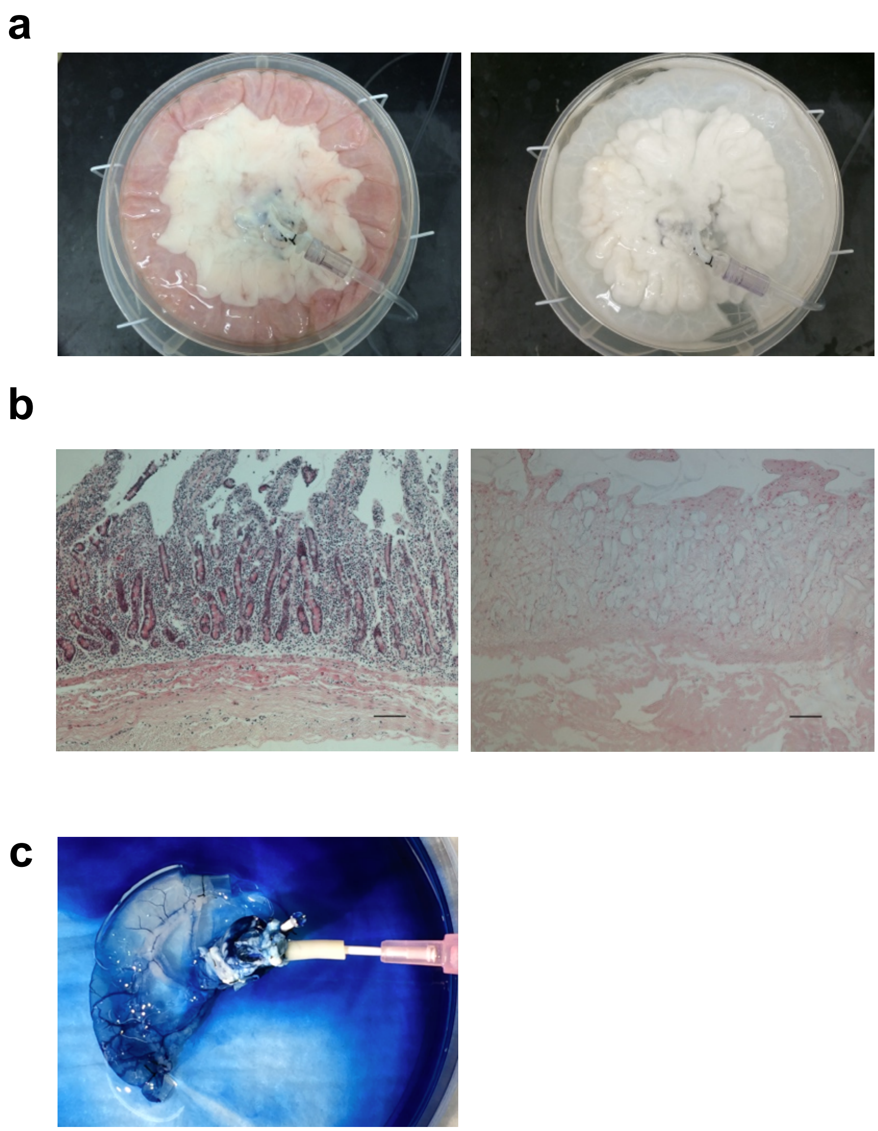

Supplementary Figure 1. Perfusion decellularization of porcine intestine. (a) Photograph of cadaveric (left) and decellularized (right) porcine jejunal segment suggesting that perfusion decellularization of rat small intestine can be upscaled to generate acellular intestinal ECMs of clinically relevant size. **(b)** Corresponding Hematoxylin and Eosin staining for cadaveric (left) and decellularized (right) intestine. Scale bars, 100 μm. **(c)** Perfusion of decellularized rat intestine with dye through the artery showing the hierarchical vascular bed was preserved.

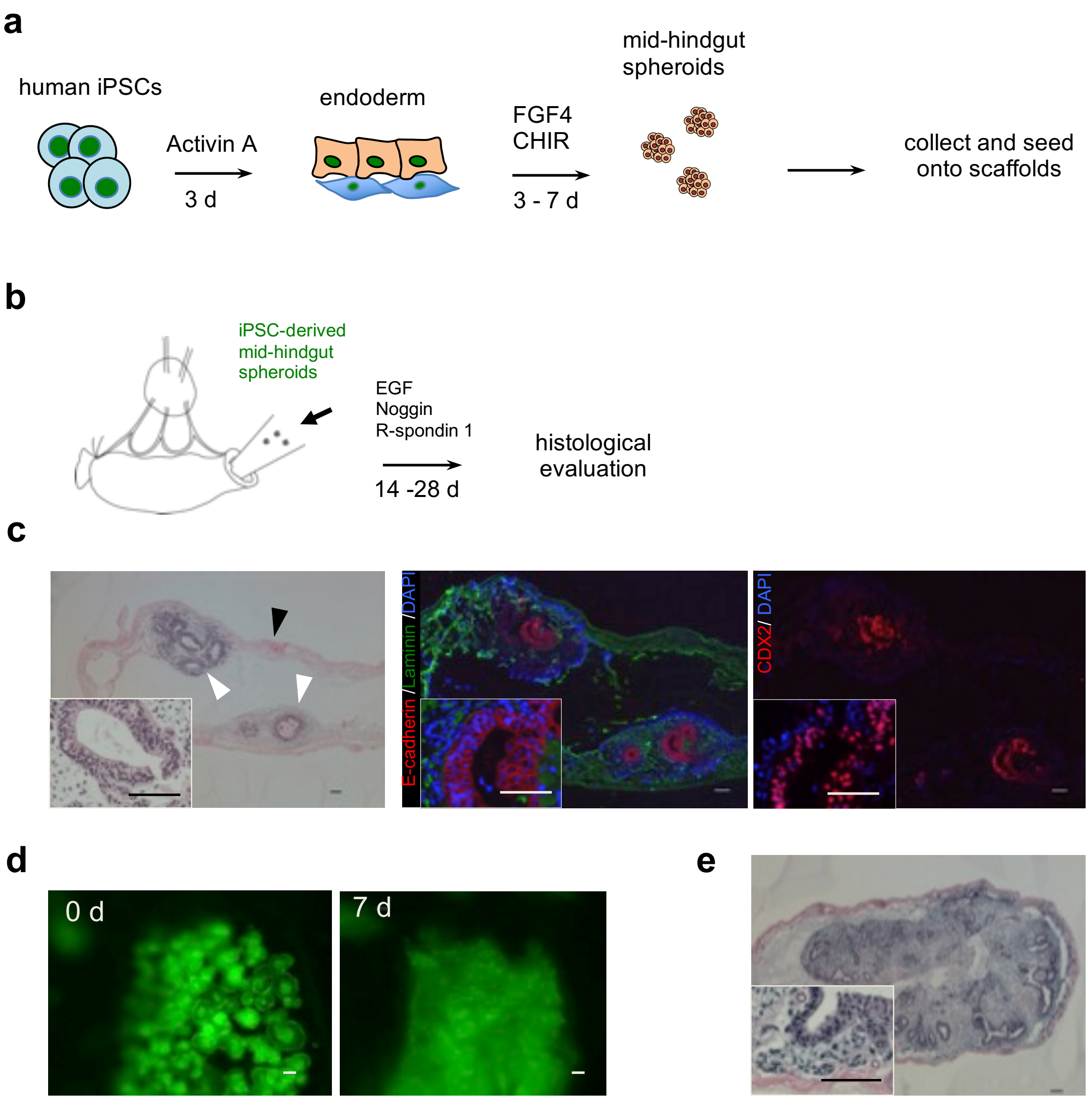

Supplementary Figure 2. Seeding of human iPSC-derived mid-hindgut spheroids on intestinal scaffold. (a) Directed differentiation of human iPSCs into mid-hindgut spheroids through definitive endoderm. **(b)** Schematic of GFP-labeled human iPSC-derived mid-hindgut spheroid seeding into a rat intestinal scaffold. Spheroid suspension was injected into the intestinal lumen. Both ends of the intestinal lumen were tied to contain the spheroids in the intestinal lumen. Further maturation into intestinal epithelium was carried out *in vitro* on a 6-well tissue culture plate. **(c)** Hematoxylin and eosin staining (left) and fluorescence micrographs (middle and right) of rat intestinal scaffold seeded with spheroids. Spheroids developed multiple epithelial-lined lumens within each site (white arrowhead) while engrafting onto the scaffold (black arrowhead). Epithelium stained positive for E-cadherin and CDX2, indicating intestinal lineage epithelial cells. Scale bars 100 μm; 10 μm in insets. **(d)** Time lapse fluorescence micrographs of GFP-labeled human iPSC-derived mid-hindgut spheroids. Spheroids merged together to form areas of continuous over 7 days of culture *in vitro*. Scale bars 100 μm. **(e)** Hematoxylin and eosin staining of rat intestinal scaffold seeded with spheroids allowed to mature *in vitro* beyond 11 days. Scale bars 100 μm.

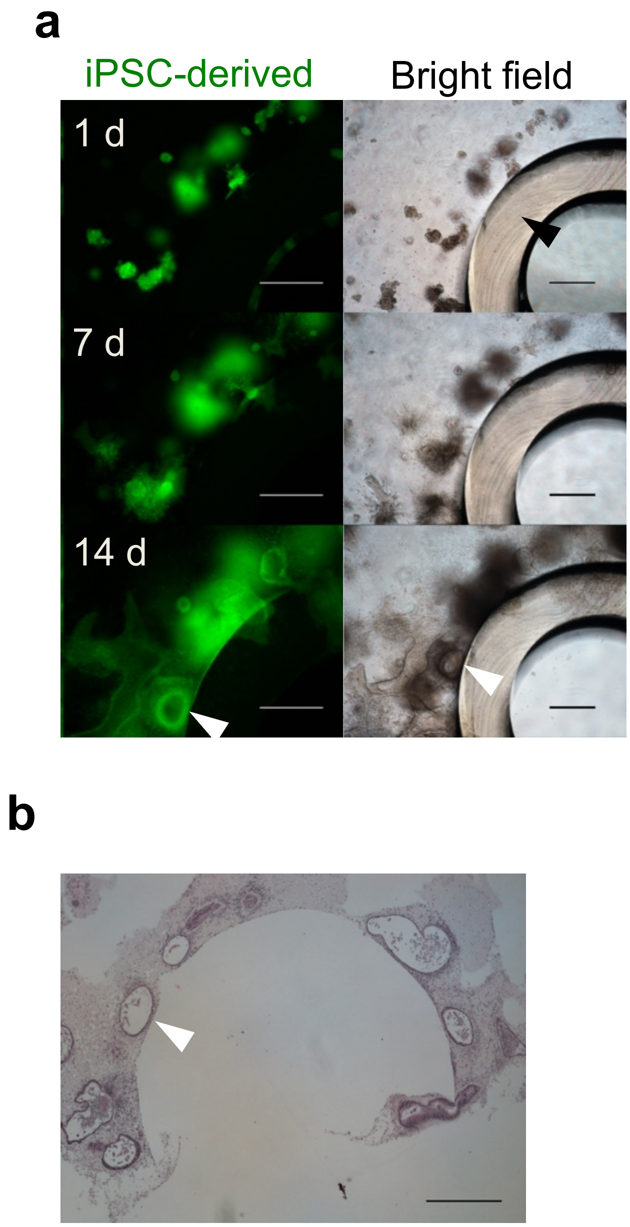

Supplementary Figure 3. ***In vitro*** **culture of spheroids grown in Matrigel with a piece of silicone tube. (a)** Time lapse fluorescence micrographs (left) and corresponding phase contrast micrographs (right) of GFP-labeled human iPSC-derived mid-hindgut spheroids grown in Matrigel. A sterile piece of silicone tube (black arrowhead) was placed on the bottom of the well. Suspension of spheroids in Matrigel was then plated such that the organoids contacted the outer surface of the silicone tube (white arrowhead). Scale bars, 500 μm. **(b)** Hematoxylin and eosin staining of the tissue grown with a piece of silicone tube for 14 days. Cells formed intestinal lumens, but did not form a continuous monolayer oriented toward the silicone tube. Scale bar, 500 μm.

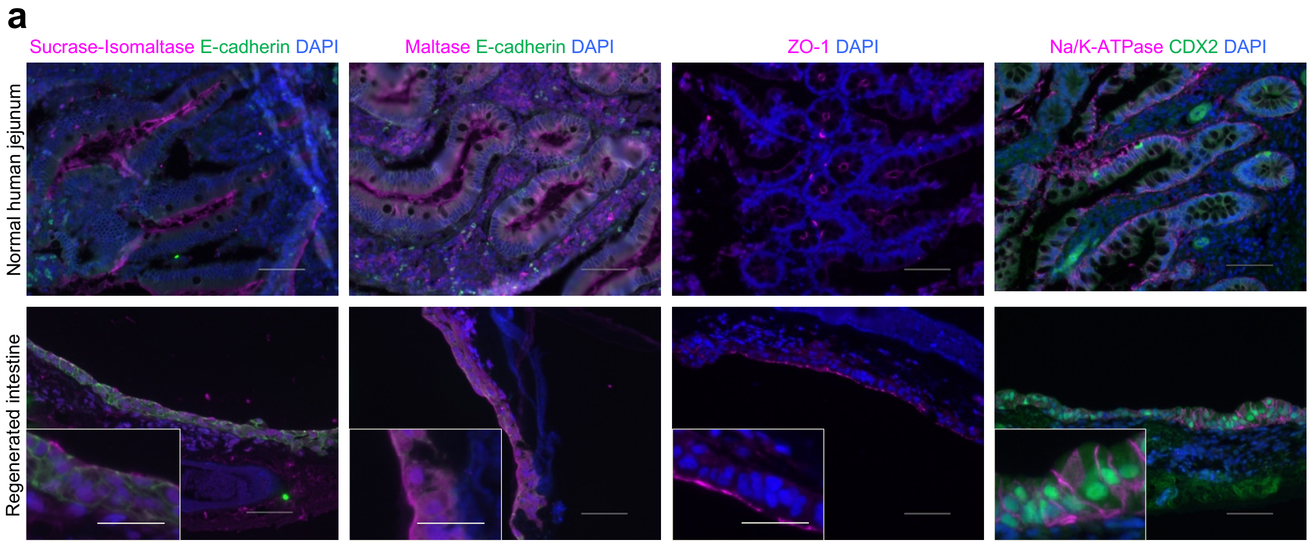

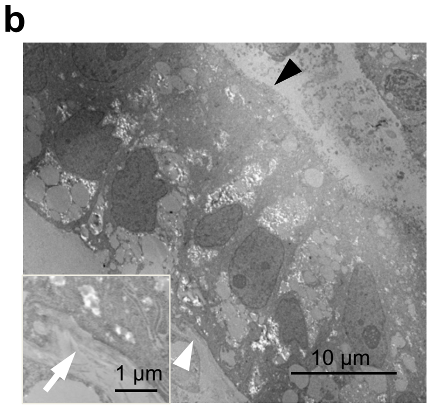

Supplementary Figure 4. Characterization of human iPSC-derived intestinal epithelium grown on decellularized intestinal scaffold. (a) Regenerated intestinal epithelium contained cells positive for Sucrase-isomaltase (left) or maltase (middle left), suggesting capacity for digestive function. Apical localization of tight junction protein ZO-1 (middle right) and basolateral expression of Na/K-ATPase (right) demonstrated polarity of the epithelial monolayer. **(b)** Transmission electron micrographs of a regenerated intestinal epithelium on rat intestinal scaffold. Monolayer of enterocyte-like cells forming apical (black arrowhead) to basolateral (white arrowhead) polarity (left). Fiber-like structures (white arrow) consistent of ECM proteins were seen (inset). Higher magnification identified presence of microvilli and cellular junctions (white arrowheads, right).

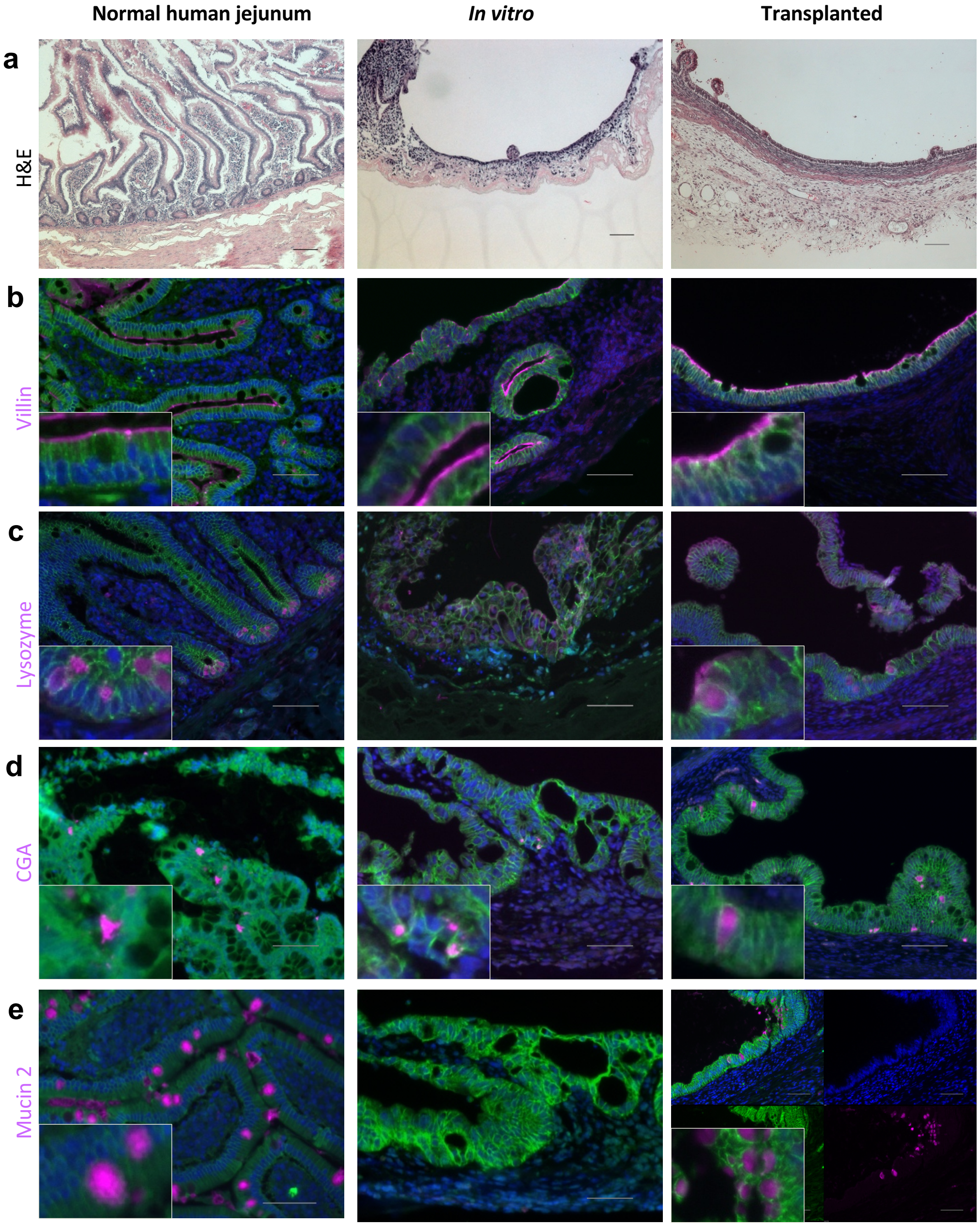

Supplementary Figure 5. Comparison of intestinal epithelial sub-types cells between native human jejunum, *in vitro* cultured recellularized scaffolds at day 28 and transplanted recellularized scaffolds at day 28. (a) H&E staining shows crypt-villous architecture of the human intestine, the *in vitro* cultured epithelium with monolayer architecture, and the transplanted epithelium with small villous projections. **(b)** Villin positively stained enterocytes in all three types of tissue. **(c)** Lysozyme stained paneth cells specifically in human intestine and transplanted epithelium, but stained many cells in a weakly positive manner in *in vitro* cultured epithelium. **(e)** Chromogranin A stained enteroendocrine cells in all three tissue types. **(f)** Mucin 2 stained goblet cells in human intestine and transplanted epithelium, but was negative in *in vitro* cultured epithelium. Insets show enlarged images for clarity in positively stained tissue. DAPI nuclear stain indicated in blue. Scale bars 100 μm.

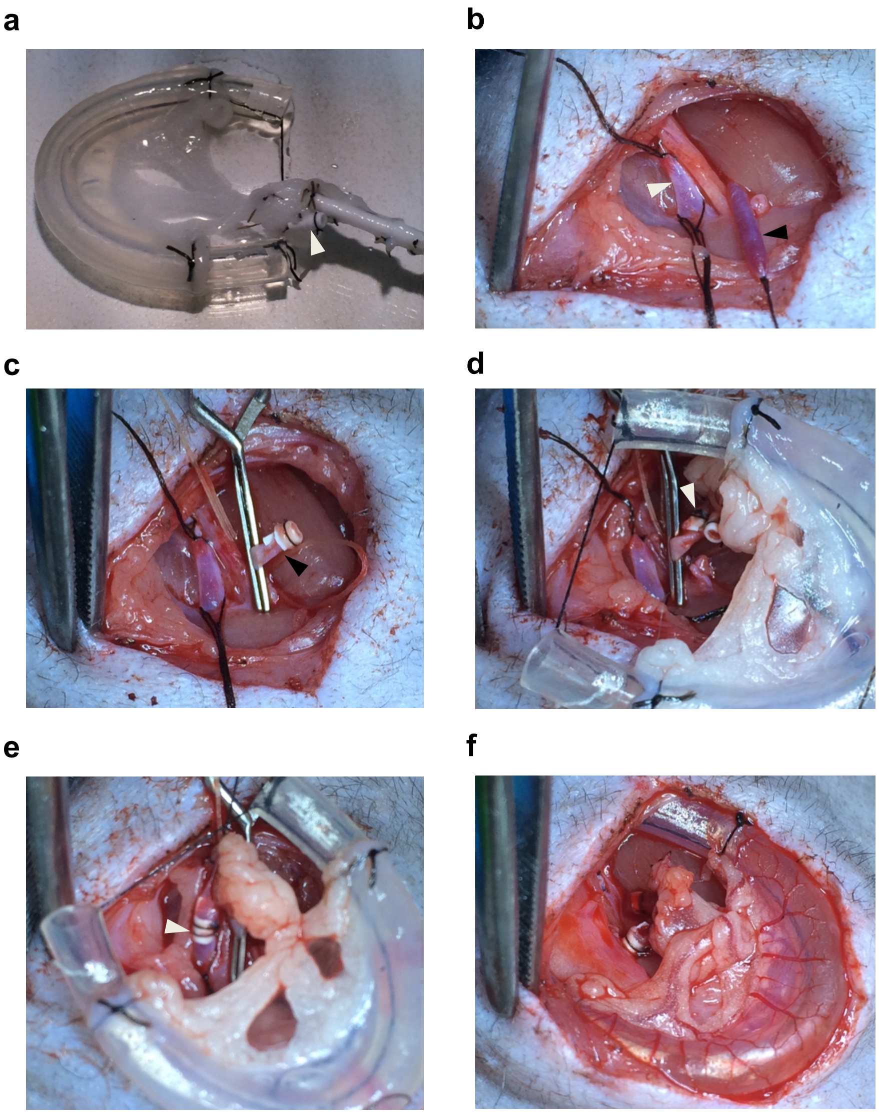

Supplementary Figure 6. Procedure of heterotopic intestinal transplantation into right cervical area of a rat. (a) Placement of a cuff on the superior mesenteric vein (SMV, white arrowhead) of the graft. **(b)** Preparation of right jugular vein (RJV, white arrowhead) and right carotid artery (RCA, black arrowhead) of the recipient. **(c)** Placement of a cuff on the RCA (black arrowhead) of the recipient. **(d)** Insertion of recipient's RCA cuff into the graft superior mesenteric artery. **(e)** Temporary ligation of the recipient's RJV. Insertion of the graft SMV cuff into the RJV (white arrowhead). **(f)** Reperfusion of the transplant graft.

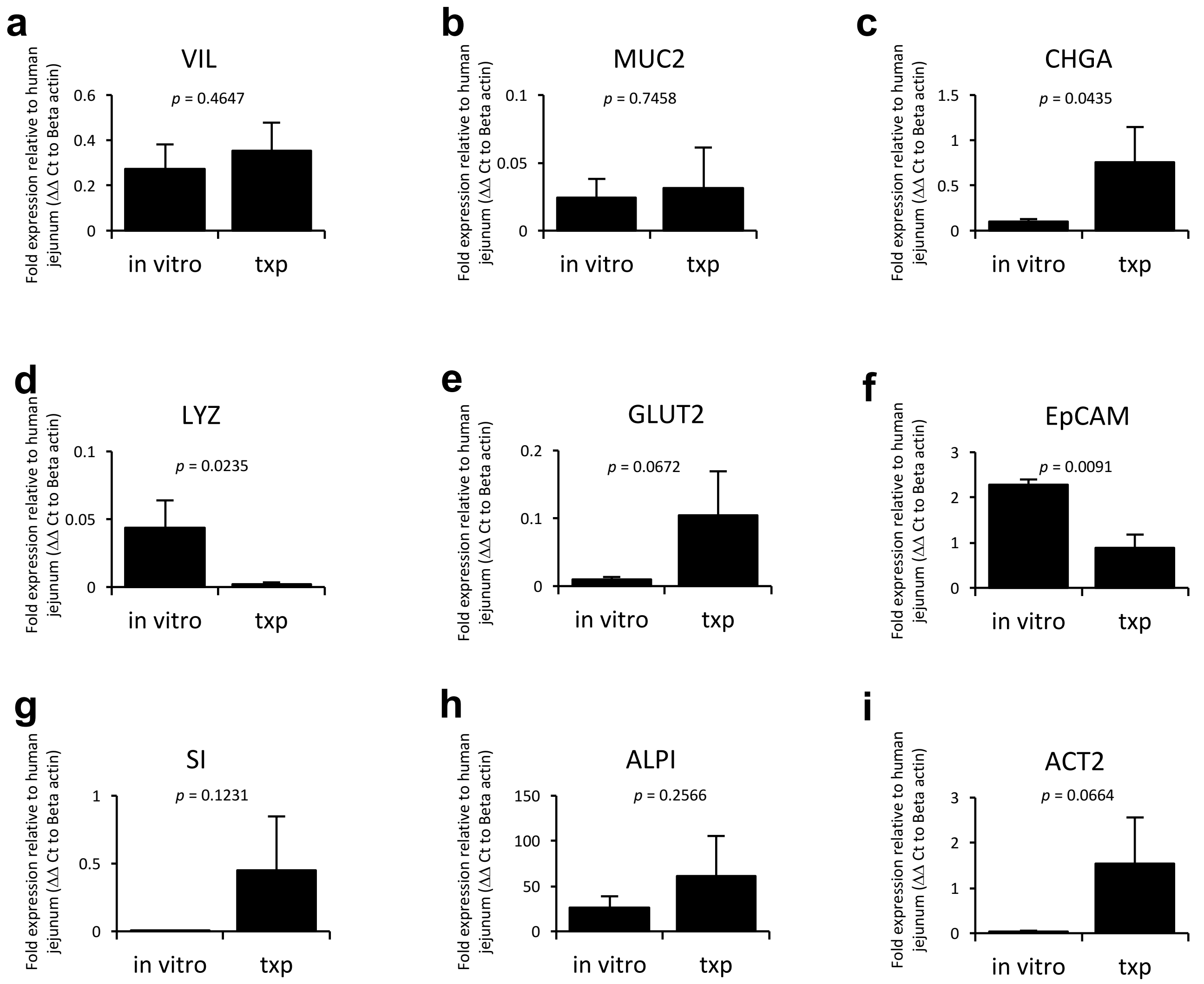

Supplementary Figure 7. Markers of epithelial and mesenchymal maturity in recellularized bowel cultured *in vitro* for 28 days versus recellularized bowel after 28 days of transplantation. (a-f) Relative gene expression of epithelial markers between *in vitro* and transplanted culture conditions, including Villin (VIL) **(a),** Mucin 2 (MUC2) **(b),** Chromogranin A (CHGA) **(c),** Lysozyme (LYZ) **(d),** and EpCam **(f).** Relative expression of intestinal enzyme markers GLUT2 **(e),** Sucrase Isomaltase (SI) **(g)** and Alkaline Phosphatase (ALPI) **(h),** as well as smooth muscle actin (α-SMA) **(i).** Graphs represent mean expression ± s.e.m, p-values for each marker are displayed. P-values calculated using one-way ANOVA.

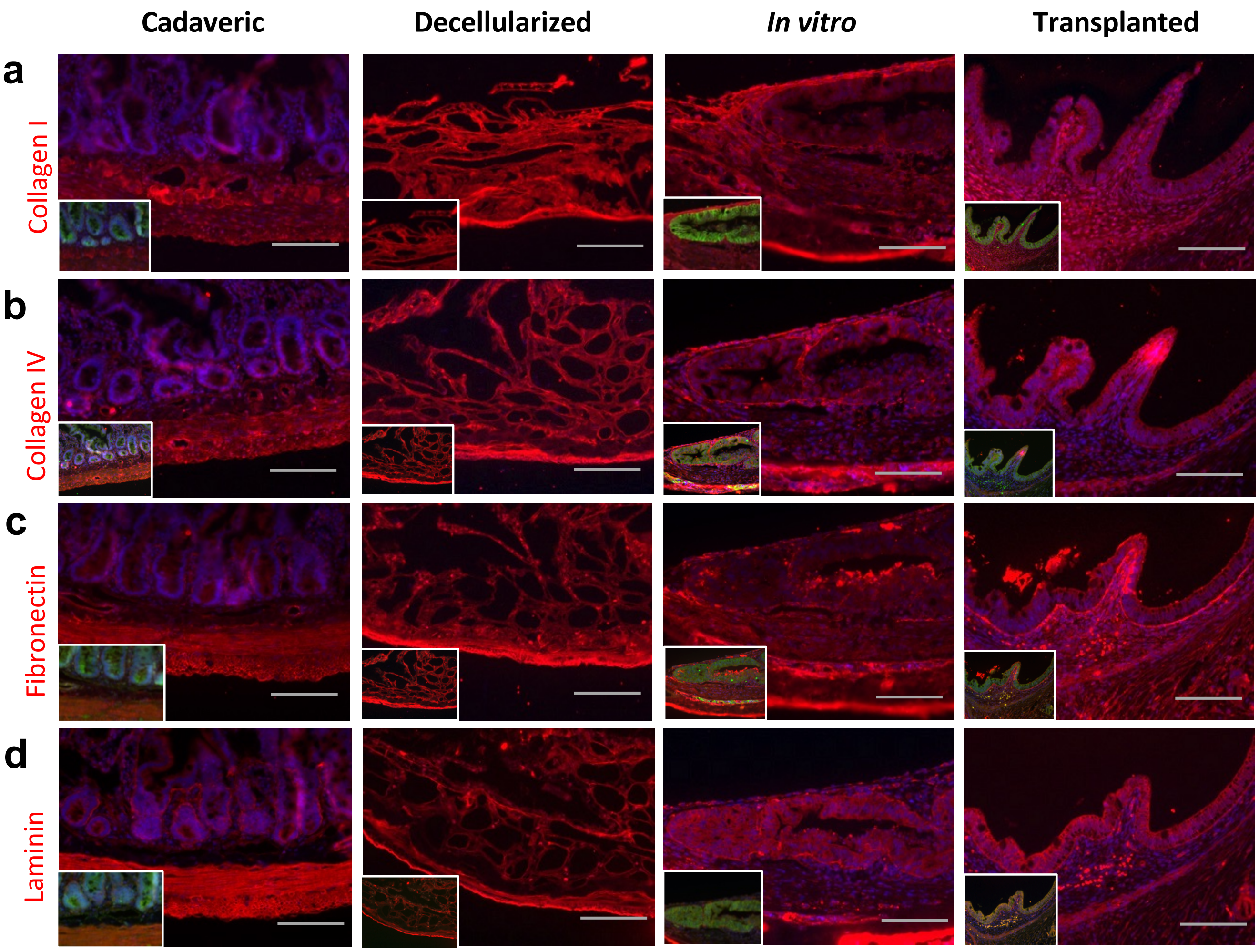

Supplementary Figure 8. Extracellular matrix component comparison. Cadaveric jejunum, decellularized scaffolds, *in vitro* recellularized scaffolds and transplanted recellularized scaffolds at 28 days were compared. **(a)** Collagen I, **(b)** collagen IV, **(c)** fibronectin and **(d)** laminin were visualized in each tissue type. DAPI nuclear staining is indicated in blue. Insets show staining for E-cadherin in green, indicative of iPSC-derived epithelium, for reference. Scale bars 100 µm.

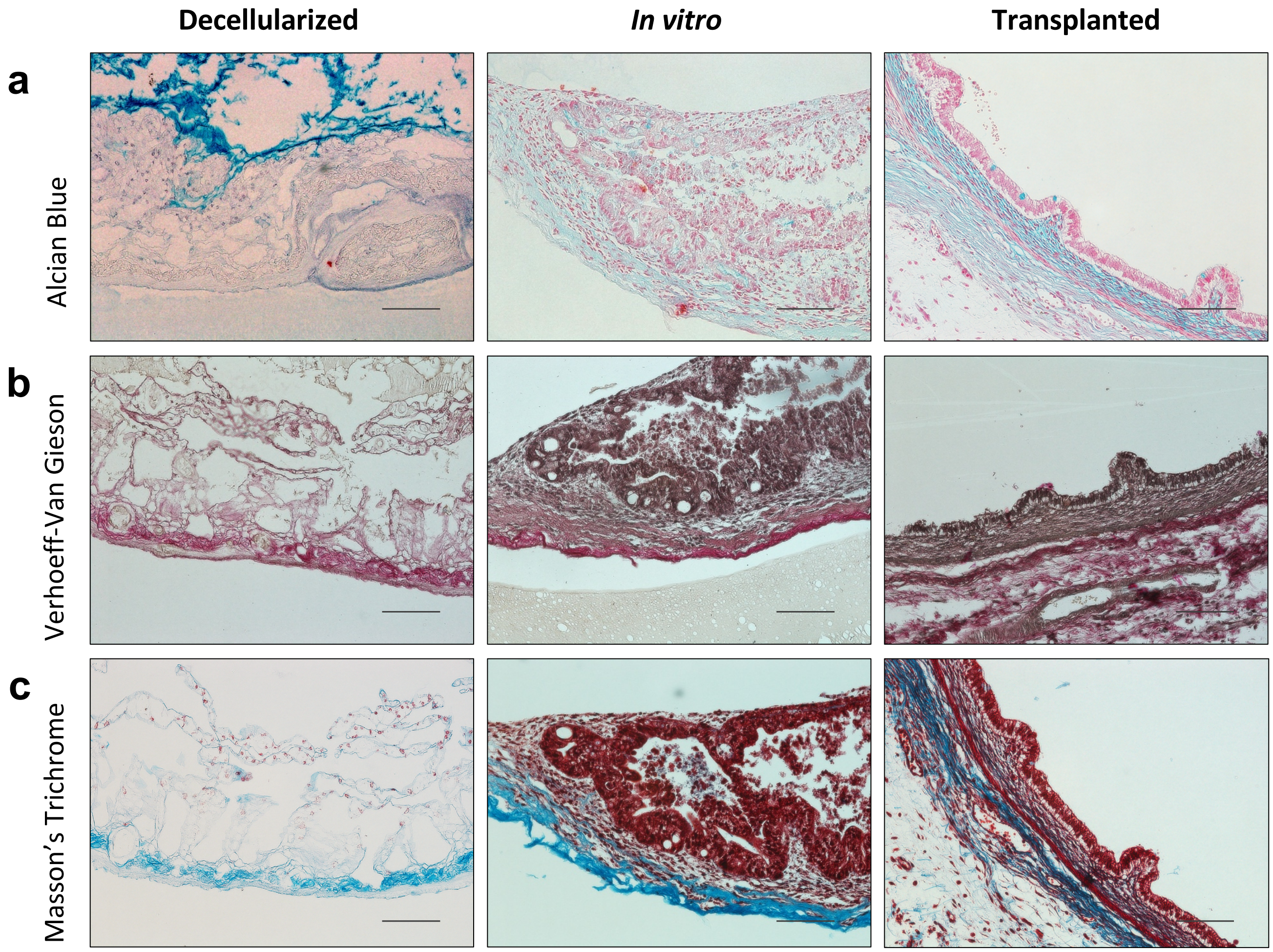

Supplementary Figure 9. Special extracellular matrix stains. A comparison of the ECM of decellularized scaffolds, *in vitro* recellularized scaffolds at day 28, and transplanted recellularized scaffolds at day 28 shows the presence of **(a)** glycosaminoglycans (blue) visualized by Alcian blue, **(b)** elastin (blue-black) visualized by Verhoeff-Van Gieson, and **(c)** collagen (blue) stained with Masson's trichrome. Scale bars 100 μm.

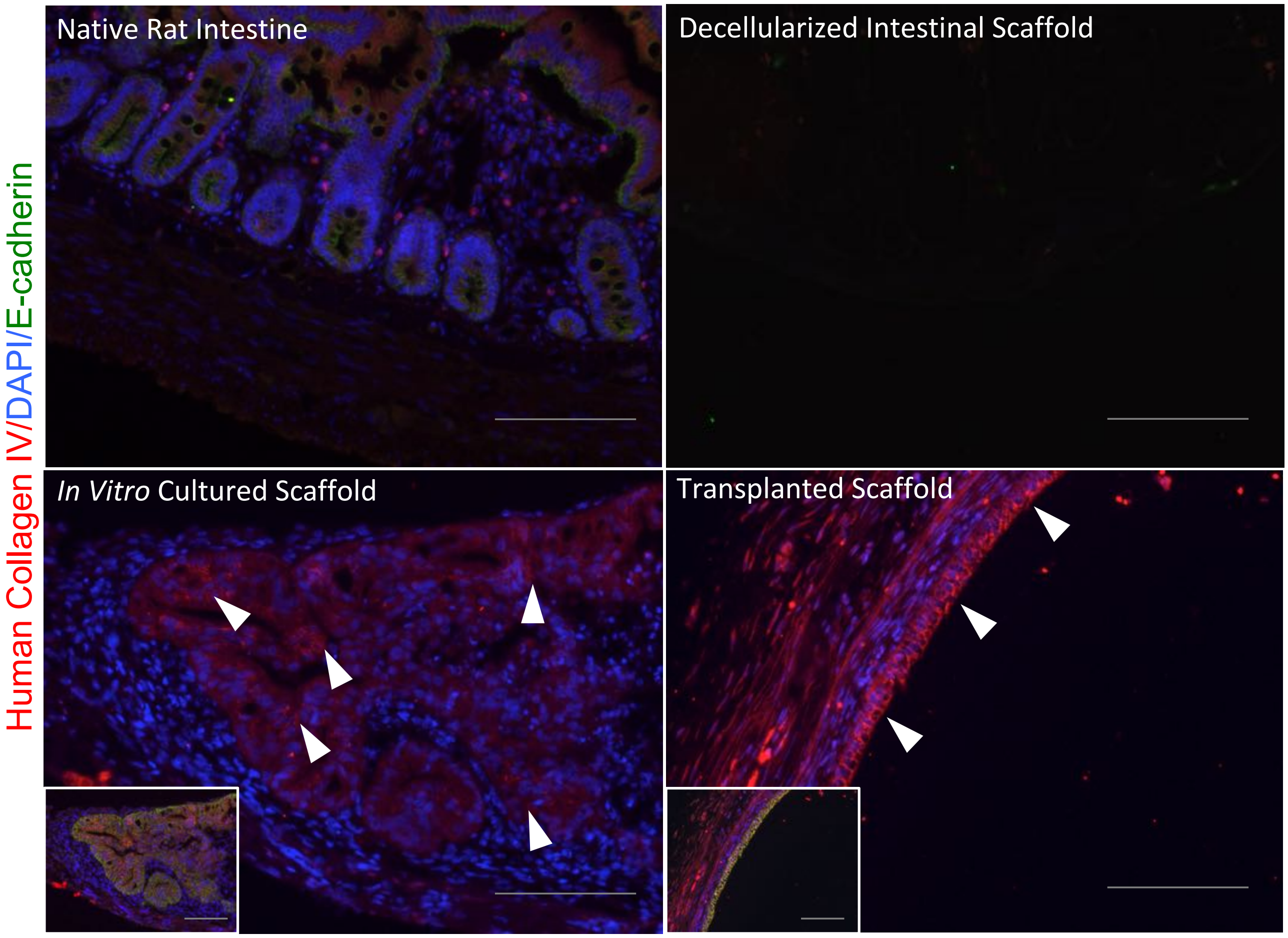

Supplementary Figure 10. iPSC-derived intestinal progenitor cells deposit collagen IV. Staining of human species-specific collagen IV in native rat intestine, a decellularized matrix scaffold, an *in vitro* cultured scaffold, and a transplanted scaffold provides evidence of ECM deposition by intestinal progenitor cells cultured on the scaffolds. Arrowheads highlight regions of human collagen IV immunoreactivity. Insets show iPSC-derived epithelium with E-cadherin stained in green for reference. Scale bars 100 μm.

Supplementary Table 1: List of primary antibodies used for immunostaining

Provider	Host	Reactivity	Name	Catalog #	Dilution factor
Abcam	Mouse	Mouse, Rat, Human	alpha 1 Na/K ATPase	ab7671	200
Abcam	Mouse	Human	alpha 5 Defensin	ab90802	50
Abcam	Rabbit	Mouse, Rat, Human	alpha SMA	ab32575	200
Dako	Mouse	Human	CD-31	M0823	200
Thermo Scientific	Rabbit	Human	CDX2	RM-2116-S0	100
Life technologies	Mouse	Human	CDX2	MA1-25822	10
Immunostar	Rabbit	Mouse, Rat, Human	Chromogranin A	20085	1000
Abcam	Rabbit	Mouse, Rat, Human	Collagen I	Ab34710	100
Abcam	Mouse	Human	Collagen IV	ab6311	100
Abcam	Rabbit	Mouse, Rat, Human	Collagen IV	ab6586	200
BD Biosciences	Mouse	Mouse, Rat, Human	E-cadherin	BD610181	200
R&D Systems	Goat	Mouse, Human	E-cadherin	AF648	100
Abcam	Rabbit	Mouse, Rat, Human	Elastin	ab21610	100
Abcam	Rabbit	Mouse, Rat, Human	Fibronectin	ab2413	200
Abcam	Rabbit	Mouse, Rat, Human	FOXA2	ab40874	1000
Abcam	Rabbit	Mouse, Rat, Human	FOXA2	ab108422	1000
Abcam	Mouse	Mouse, Rat, Human	FOXA2	ab60721	1000
Santa-Cruz	Goat	Mouse, Rat, Human	Gastric Inhibitory Peptide	sc-23554	200
proteintech	Rabbit	Mouse, Rat, Human	GLUT2 (SLC2A2)	20436-1-AP	100
Millipore	Mouse	Human	Human Mitochondria	MAB1273	200
Millipore	Mouse	Human	Human Nuclear	MAB1281	100
Abcam	Rabbit	Mouse, Rat, Human	Ki67	Ab16667	100
Abcam	Rabbit	Human	Lamin A+C	ab108595	200
Abcam	Rabbit	Mouse, Rat, Human	Laminin	ab11575	200
Abcam	Mouse	Human	Lysozyme	ab36362	150
Dako	Rabbit	Human	Lysozyme	A0099	200
Santa-Cruz	Rabbit	Mouse, Rat, Human	Maltase-glucoamylase	sc-98598	100
Abcam	Rabbit	Rat, Human	MUC2	ab134119	200
Santa-Cruz	Rabbit	Mouse, Rat, Human	Mucin 2	sc-15334	150
Santa-Cruz	Goat	Mouse, Rat, Human	Oct-3/4	sc-8628	200
Santa-Cruz	Rabbit	Mouse, Human	PepT1 (SLC15A1)	sc-20653	200
Bio-Rad Serotec	Mouse	Rat	Rat CD68	MCA341R	100
R&D Systems	Goat	Human	SOX17	AF1924	1000
Abcam	Mouse	Mouse, Rat, Human	αSMA	Ab7817	100
Santa-Cruz	Goat	Mouse, Rat, Human	Sucrase-Isomaltase	sc-27603	100
Abcam	Rabbit	Human	Villin	ab52102	50
Proteintech	Rabbit	Mouse, Rat, Human	Villin 1	16488-1-AP	150
R&D Systems	Rat	Mouse, Rat, Human	Vimentin	280618	100
Abcam	Rabbit	Rat, Human	Von Willebrand Factor	ab6994	200
Life technologies	Rabbit	Mouse, Rat, Human	ZO-1 / TJP1	61-7300	100

Supplementary Table 2: List of secondary antibodies used for immunostaining

Provider	Label	Host	Reactivity	Name	Catalog #	Dilution Factor
Life Technologies	AF350	Donkey	Goat	anti-Goat IgG (H+L)	A-21081	500
Life Technologies	AF405	Goat	Mouse	anti-Mouse IgG (H+L)	A-31553	500
Life Technologies	AF488	Donkey	Rabbit	anti-Rabbit IgG (H+L)	A-21206	500
Life Technologies	AF488	Donkey	Mouse	anti-Mouse IgG (H+L)	A-21202	500
Life Technologies	AF488	Donkey	Goat	anti-Goat IgG (H+L)	A-11055	500
Life Technologies	AF546	Donkey	Rabbit	anti-Rabbit IgG (H+L)	A-10040	500
Life Technologies	AF546	Donkey	Mouse	anti-Mouse IgG (H+L)	A-10036	500
Life Technologies	AF546	Donkey	Goat	anti-Goat IgG (H+L)	A-11056	500
Life Technologies	AF594	Donkey	Mouse	anti-Mouse IgG (H+L)	A-21203	500
Life Technologies	AF594	Donkey	Rabbit	anti-Rabbit IgG (H+L)	A-21207	500
Life Technologies	AF594	Donkey	Goat	anti-Goat IgG (H+L)	A-11058	500
Life Technologies	AF647	Goat	Rabbit	anti-Rabbit IgG (H+L)	A-21244	500
Life Technologies	AF647	Donkey	Mouse	anti-Mouse IgG (H+L)	A-31571	500
Life Technologies	AF647	Donkey	Goat	anti-Goat IgG (H+L)	A-21447	500
Life Technologies	AF647	Donkey	Rabbit	anti-Rabbit IgG (H+L)	A-31573	500
Life Technologies	AF488	Donkey	Rat	anti-Rat IgG (H+L)	A-21208	500
Life Technologies	Cy® 5	Goat	Mouse	anti-Mouse IgG (H+L)	A-10524	500
